# Synthesis and Evaluation of a Novel Zuranolone Analog with High GABA_A_ Receptor PAM Activity and Excellent Pharmacokinetic Profiles

**DOI:** 10.3390/molecules30091918

**Published:** 2025-04-25

**Authors:** Yingjie Yang, Xu Deng, Hengwei Xu, Daoyuan Chen, Fengjuan Zhao, Huijie Yang, Wenyan Wang, Chunjie Sha, Mingxu Ma, Guanqing Zhang, Liang Ye, Jingwei Tian

**Affiliations:** 1 Key Laboratory of Molecular Pharmacology and Drug Evaluation, School of Pharmacy, Ministry of Education, Collaborative Innovation Center of Advanced Drug Delivery System and Biotech Drugs in Universities of Shandong, Yantai University, Yantai 264005, China; 2 State Key Laboratory of Advanced Drug Delivery and Release Systems, Shandong Luye Pharmaceutical Co., Ltd., Yantai 264003, China; 3 Department of Traditional Chinese Medicine, Shandong College of Traditional Chinese Medicine, Yantai 264119, China; 4 School of Pharmacy, Binzhou Medical University, Yantai 256603, China; 5 School of Public Health and Management, Binzhou Medical University, Yantai 256603, China

**Keywords:** Zuranolone analog, neurosteroid (NAS), GABA_A_ receptors, positive allosteric modulator (PAM), lipophilicity, anticonvulsant effect, pharmacokinetic evaluation

## Abstract

Zuranolone (SAGE-217), the first FDA-approved oral neurosteroid (NAS), a positive allosteric modulator (PAM) of γ-aminobutyric acid type A (GABA_A_) receptor for postpartum depression approved in 2023, has limitations such as short half-life, low bioavailability, and central inhibitory side effects. To address these, we designed novel C-21 modified derivatives of Zuranolone, identifying the triazolone scaffold as key for enhancing GABA_A_ activity. Here, we synthesized Zuranolone analogs with diverse triazolone substituents, finding that pyridine-derived modifications improved the activity correlated with LogP. The optimal derivative, **S9** (2-(trifluoroethoxy)pyridine-triazolone, LogP 4.61), showed 2.5-fold greater potency (EC_50_) and efficacy (Emax) than Zuranolone (LogP 4.78) at synaptic/extrasynaptic GABA_A_ receptors, attributed to stronger binding via molecular docking. In rats, **S9** exhibited 5-fold longer plasma T_1/2_, 6-fold higher AUC, 3-fold greater brain exposure, and 30% improved bioavailability. It also outperformed Zuranolone in pentylenetetrazole (PTZ)-induced seizure suppression and threshold dose for loss of righting reflex (LORR) in rats. The C21-pyridine-triazolone pharmacophore in **S9** enhances receptor activity potency without increasing lipophilicity, optimizing pharmacokinetics and safety, which makes it a promising therapeutic candidate for depression and epilepsy.

## 1. Introduction

Neurosteroids (NASs), such as endogenous allopregnanolone (AlloP; brexanolone), act as positive allosteric modulators (PAMs) for γ-aminobutyric acid type A (GABA_A_) receptors, regulating neuronal excitability [1]. GABA_A_ receptor subtypes consisting of α1-3, β1-3, and γ2 subunits are primarily positioned at synapses, mediating rapid phasic inhibition, while those consisting of α4-6, β2/3, and δ subunits are predominantly located at extrasynaptic sites, mediating sustained tonic inhibition [1,2]. GABA_A_ receptors are essential in regulating the functions of mental and behavioral networks, and the downregulation of NAS synthesis and dysfunction of GABA_A_ receptors have been linked to the pathogenesis of neuropsychiatric disorders, including depression and epilepsy [2,3,4,5]. Thus, GABA_A_ receptors are critical targets for barbiturates, benzodiazepines, anesthetics, and analgesics, as well as for the development of novel drugs [3,4,5,6]. Classical benzodiazepines only allosterically potentiate synaptic γ-containing GABA_A_ receptors. In contrast, NASs exert their effects by allosterically modulating and directly activating all GABA_A_ receptor subtypes [2]. Moreover, NASs increase the surface expression and phosphorylation of δ-containing extrasynaptic GABA_A_ receptors [7,8]. The unique pharmacological properties of NASs prevent the development of tolerance upon repeated use in epileptic disorders and enable rapid antidepressant effects, indicating a significant advantage over benzodiazepines [3,4,5,6].

**Figure 1:**
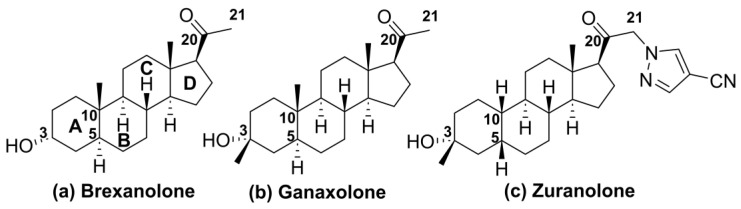
Chemical structures of neurosteroid agents. A, B, C, D: A, B, C and D rings of the steroidal structure.

The approved NAS drugs (Figure 1) function as powerful and highly effective PAMs targeting GABA_A_ receptors. Structure–activity relationship (SAR) studies of NASs indicate that the 3α-hydroxyl and C20-ketone groups are critical sites for allosteric potentiation by pregnane-derived NASs [9,10,11]. Additionally, the C20 branch (C21 position) group contributes to coordinating the affinity with the hydrophobic pocket, thereby influencing both potency and efficacy [12]. Zulresso (SAGE-547) is an intravenous formulation of AlloP (Figure 1a) for treating postpartum depression (PPD). The limited bioavailability of natural AlloP results from its rapid hepatic metabolism, involving oxidation of the C3-hydroxyl group and glucuronide conjugation [13,14]. Ztalmy (Ganaxolone; Figure 1b), an orally administered synthetic analog of AlloP, is an antiepileptic drug that exhibits increased bioavailability due to the introduction of a 3β-methyl group, which inhibits the rapid metabolic inactivation of the C3-hydroxy group in the liver [14]. Another orally active agent for PPD, Zurzuvae (Zuranolone, SAGE-217; Figure 1c), introduces a 4-cyanopyrazole substituent at the C21 position, which significantly enhances activity at GABA_A_ receptors and improves bioavailability [15,16,17]. However, Zuranolone has notable limitations, including a ‘black box’ warning for its potential to impair cognitive and motor functions, such as driving or operating machinery, along with risks of central nervous system (CNS) depression and embryo-fetal toxicity [18,19]. In addition, Zuranolone and Ganaxolone (GX), as synthetic neuroactive steroids, still exhibit some of the inherent biopharmaceutic drawbacks of natural NASs to varying degrees, such as poor aqueous solubility, food effects, rapid hepatic metabolism, and low bioavailability, thereby limiting their clinical therapeutics [14,16,18,19,20,21,22]. Therefore, some studies have focused on developing potential new drugs for depression and epilepsy disorders by modifying the structures of marketed NAS drugs to overcome these limitations [22,23,24,25].

Recently, our study on the C21-substituents of Zuranolone has shown that the triazolone scaffold exhibits optimal structural characteristics for GABA_A_ receptor activity [25], and its derivative has comparable anticonvulsant effects to GX [26]. Since NASs are highly lipophilic molecules with high LogP values, the nonspecific lipophilicity of the molecules and the characteristics of specific pharmacophore substituents significantly affect not only their potency and efficacy but also their biopharmaceutic properties [27,28,29]. Therefore, based on our previous research, we are now primarily attempting to further modify and optimize triazolone derivatives by introducing substituents with diverse structures and electronic properties. Furthermore, we aim to study the effects of structure and lipophilicity on activity and pharmacokinetic properties to identify more potent PAMs of GABA_A_ receptors.

The results showed that replacing the pyrazole group at the C21 position of Zuranolone with trifluoroethoxypyridine-triazolone (**S9**) leads to a greater potency for GABA_A_ receptors, excellent pharmacokinetic profiles, and a broader safety window, but without increased lipophilicity. The research results support its potential as a novel candidate drug of NASs for treating depression and/or epilepsy, which warrants further investigation.

## 2. Results

### 2.1. Synthesis Overview

**Scheme 1:**
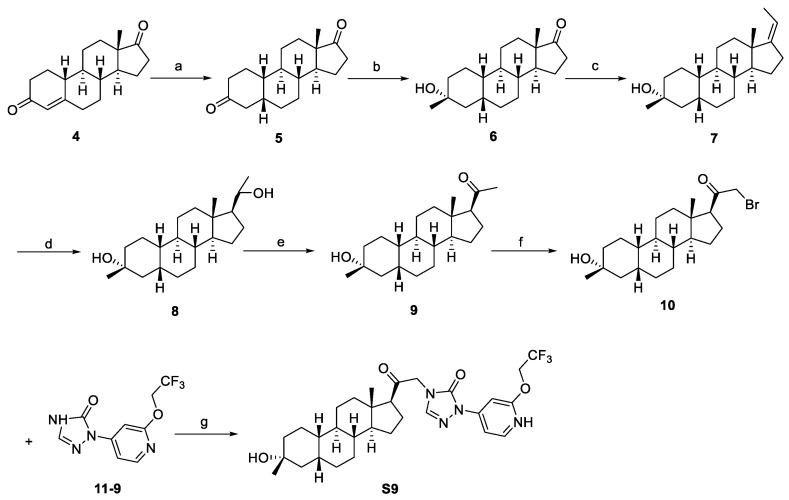
Synthetic routes of compound **S9**: reagents and conditions. (**a**) 10%Pd/C, H_2_, HBr, THF, 25 °C, 24 h, crude; (**b**) MAD, MeMgBr, Toluene, THF, −60 °C, 3 h, yield% = 41% for two steps; (**c**) t-BuOK, EtP^+^PhBr^−^, THF, 0–60 °C, 13 h, yield% = 88%; (**d**) BH_3_·Me_2_S, NaOH·H_2_O_2_ THF, 0–25 °C, 4 h, yield% = 67%; (**e**) PCC, DCM, 25 °C, 3 h, yield% = 70%; (**f**) HBr, Br_2_, MeOH, 25 °C, 12 h, yield% = 63%; (**g**) K_2_CO_3_, THF, 50 °C, 12 h, Yield% = 63%.

**Scheme 2:**
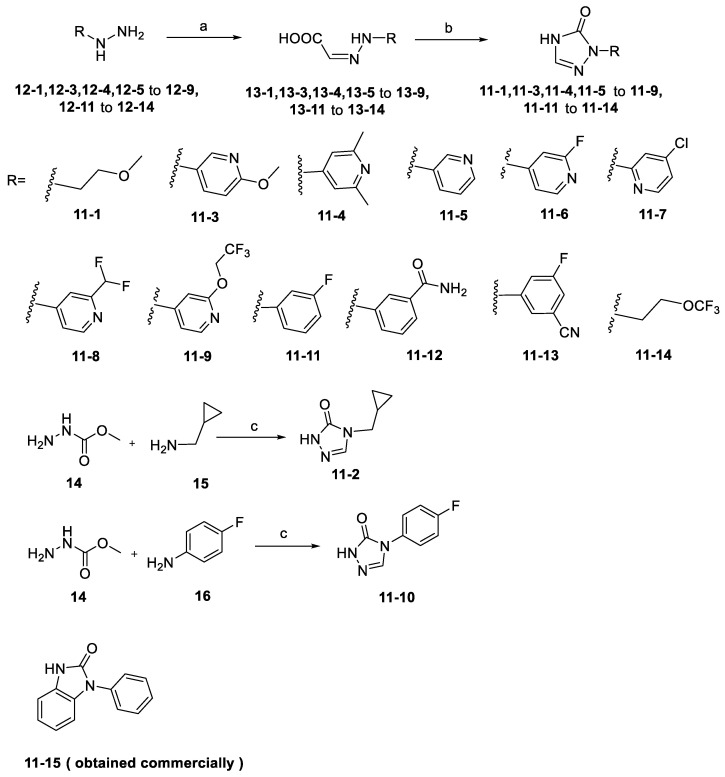
Reagents and reaction conditions: (**a**) oxaldehydic acid, 25 °C, HCl/H_2_O, 16 h, 52.8%; (**b**) diphenylphosphoryl azide, TEA, 90 °C, 16 h, 43.1%; (**c**) trimethoxymethane, MeONa, MeOH, 70 °C, 13 h, 7%.

A series of analogs of compound **S9** and relative intermediates were synthesized, and the synthetic routes are shown in Scheme 1 and Scheme 2.

Starting from commercially available material **4**, intermediate **5** was prepared via 10 wt% Pd/C hydrogenation reduction. The synthesis of intermediate **6** involves addressing the chemoselectivity and stereoselectivity challenges associated with the introduction of 3β-methyl groups. Previous literature has reported the use of methylaluminum bis(2,6-di-tert-butyl-4-methylphenoxide) (MAD) in combination with Grignard reagents to control both the chemical and stereoselective introduction of methyl groups [16]. Accordingly, we adopted the same method reported in the literature, successfully synthesizing intermediate **6** through a two-step procedure with an overall yield of 41%. Intermediate **6** underwent a Wittig reaction to yield α, β-unsaturated ester **7** with a yield of 88%. Subsequently, intermediate **7** was employed as the starting material to generate intermediate **9** through a two-step reaction sequence comprising a hydroboration–oxidation reaction followed by a pyridinium chlorochromate (PCC)-mediated oxidation, with isolated yields of 67% and 70%, respectively. Then, intermediate **9** was brominated to obtain brominated intermediate **10**. The target compound **S9** was finally synthesized from brominated intermediate **10** and compound **11-9** through an SN2 substitution reaction using K_2_CO_3_ as base (Scheme 1).

The different hydrazine imide intermediates (**13-1**, **13-3**, **13-4**, **13-5** to **13-9**, and **13-11** to **13-14**) were synthesized via a condensation reaction between substituted alkyl-hydrazines or aryl-hydrazines (**12-1**, **12-3**, **12-4**, **12-5** to **12-9**, and **12-11** to **12-14**) and glyoxylic acid. Subsequently, the carboxyl group of hydrazine imide intermediates reacted with diphenylphosphoryl azide to form an acyl azide intermediate, which can react with intramolecular amine cyclization to yield triazole derivative intermediates (**11-1**, **11-3**, **11-4**, **11-5** to **11-9**, and **11-11** to **11-14**). The tetrazole intermediate **11-2** was generated through a two-step reaction: first, methyl hydrazinecarboxylate (**14**) and trimethoxymethane reacted to generate an active intermediate, which reacted with cyclopropylmethanamine (**15**) through an intramolecular SN2 reaction under alkaline conditions with MeONa. The intermediate **11-10** was then synthesized by replacing cyclopropylmethanamine (**15**) with 4-fluoroaniline (**16**) using the same reaction procedure as **11-2**. The tetrazole intermediate **11-15** was obtained commercially. The target compounds **S1**–**S8** and **S10**–**S15** were synthesized by the same method as **S9** (Scheme 2).

### 2.2. Physicochemical Parameters of Zuranolone and Synthetic Compounds

ADMETlab3.0 software [30] was utilized to predict the physicochemical parameters of Zuranolone and its analogs, as shown in Table 1. **S9** and Zuranolone exhibited similar pKa ranges, but **S9** demonstrated a lower LogP (4.61 vs. 4.78), a reduced LogD (4.13 vs. 4.39), and a higher topological polar surface area (TPSA) (99.2 Å^2^ vs. 78.9 Å^2^) compared to Zuranolone. These differences suggest that **S9** has an improved solubility profile, which could enhance its pharmacokinetic properties and overall drug-like characteristics.

### 2.3. Preliminary Structure–Activity Relationship Study

The α1β2γ2 and α4β3δ GABA_A_ receptor subtypes, representing synaptic and extrasynaptic subunit compositions in neurons, are widely utilized for screening new NAS drugs [2,16]. In this study, a preliminary SAR study was conducted on a series of Zuranolone analogs using the LogP values predicted by ADMETlab3.0 software, recombinant human GABA_A_ receptor cell lines, and in vitro liver microsome stability assays. The results are summarized in Table 1, Table 2, and Table 3, respectively.

Our previous results showed that replacing the C21 pyrazole group of Zuranolone with triazolone significantly reduces its potency (EC_50_) and efficacy (E_max_) at synaptic GABA_A_ receptors to less than 35% of Zuranolone, and to less than 50% at extrasynaptic GABA_A_ receptors [25]. Several analogs of Zuranolone have been synthesized by substituting the pyrazole group at the C21 position with triazolone derivatives. These analogs were prepared by introducing groups with different structures and electronic properties onto the nitrogen atom at the 2-position of triazolone. Research results on electron-donating pyridine derivatives indicate that the activities of the 2-methoxypyridine derivative (**S3**) and the 2,6-dimethylpyridine derivative (**S4**) are both lower than 50% of that of Zuranolone. Further research on electron-withdrawing pyridine derivatives revealed that the activities of the pyridine derivative (**S5**) and the 2-fluoropyridine derivative (**S6**) show no significant improvement compared to **S3** or **S4**. The para-chloropyridine derivative (**S7**) exhibited slightly higher potency at synaptic and extrasynaptic GABA_A_ receptor subtypes, approximately 10% higher than Zuranolone, with efficacy reaching 70% of that of Zuranolone. Moreover, increasing the number of electronegative elements on the pyridine ring demonstrated that the potency of the 2-(difluoromethyl)pyridine derivative (**S8**) at synaptic and extrasynaptic GABA_A_ receptors is 140% and 150% of that of Zuranolone, respectively, with an efficacy of about 60%. The potency of the 2-(trifluoroethoxy)pyridine derivative (**S9**) at synaptic and extrasynaptic GABA_A_ receptors is 250% and 270% of that of Zuranolone, respectively, with efficacies of 76% and 71%. Computational results indicate that the LogP values of **S9**, **S8**, **S6**, and **S5** are 4.61 > 3.88 > 3.70 ≈ 3.78, respectively, which is basically consistent with the order of their receptor activity levels. Moreover, benzene derivatives with different electronegative elements, including the para-fluorobenzene derivative (**S10**), the meta-fluorobenzene derivative (**S11**), and the benzamide derivative (**S12**), exhibited relatively low activity at all GABA_A_ receptor subtypes, mostly less than 50% of that of Zuranolone. Although the 3-cyano-5-fluorobenzene derivative (**S13**) showed increased potency, comparable to that of **S8**, no significant improvement in efficacy was observed.

The above results suggest that introducing 2-(difluoromethyl)pyridine (**S8**) and 2-(trifluoroethoxy)pyridine (**S9**) onto triazolone enhances the effects on both synaptic and extrasynaptic GABA_A_ receptors, which is consistent with their relatively high LogP values. Furthermore, in vitro liver microsome stability studies showed that **S8** is less stable, whereas **S9** exhibits high stability. Based on these results, **S9**, which shows the highest potency and high metabolic stability, was selected as a candidate compound for further research.

### 2.4. Molecular Docking Analysis

**Figure 2:**
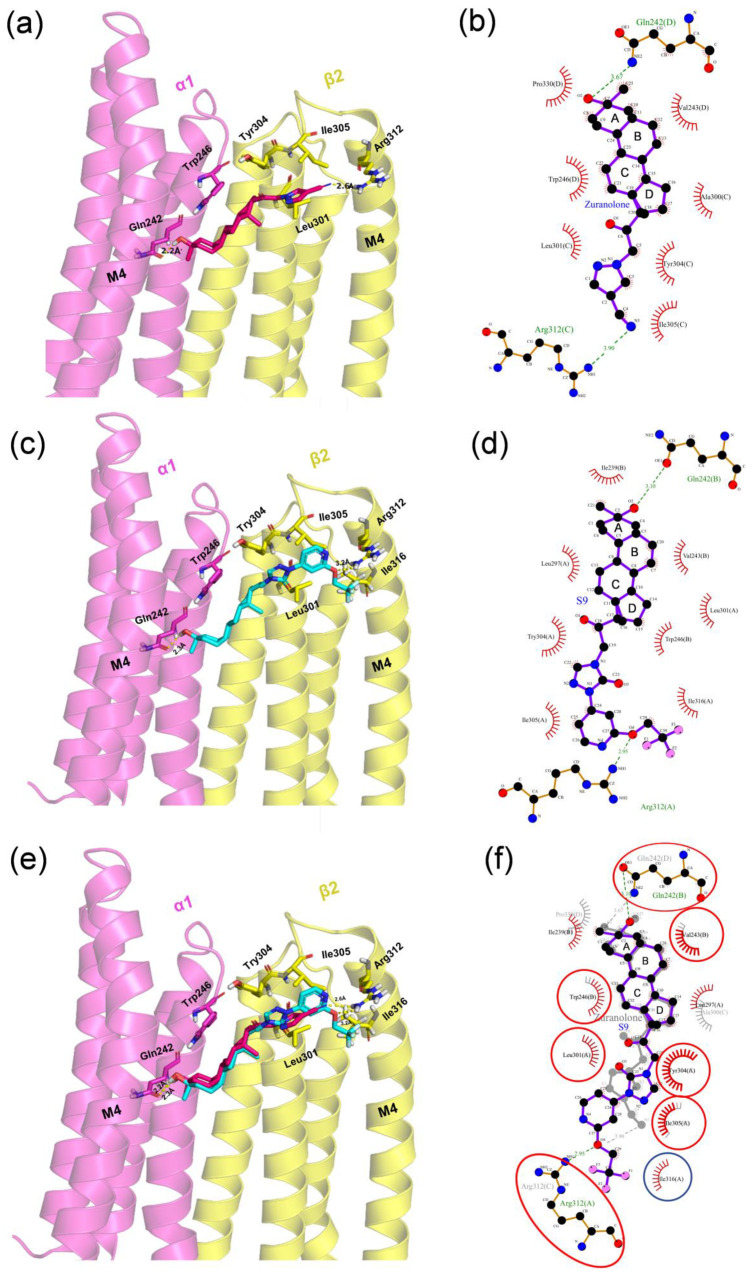
Docking modes of Zuranolone and **S9** with α1β2γ2 GABA_A_ receptor subunits (PDB: 8SI9). Subunits are colored in magenta (α1) and yellow (β2). The 3D docking diagrams of Zuranolone (red) (**a**), **S9** (cyan) (**c**), and the overlap of them (**e**) with the GABA_A_ receptor were constructed with Maestro software (version 2018, Schrödinger LLC, New York, NY, USA). The interactions between ligands and residues are represented by the dashed lines. The 2D docking diagrams of Zuranolone (**b**), **S9** (**d**), and the overlap of them (**f**) with the GABA_A_ receptor were created using Ligplot (version 2.2.8, EMBL-EBI, Hinxton, Cambridgeshire, UK). A, B, C, D: A, B, C and D rings of the steroidal structure.

Studies utilizing the cocrystal structure of AlloP with the GABA_A_ receptor α1β2γ2 subunit have demonstrated that AlloP binds to the β-α interfaces within the transmembrane domain, a recognized binding site for NASs [31]. Docking results revealed that Zuranolone and **S9** share similar molecular orientations in the binding pocket but exhibit key differences in interactions. The C-3 tertiary hydroxyl groups of both compounds form hydrogen bonds with α1-GLN242, with bond lengths of 2.2 Å and 2.3 Å for Zuranolone and **S9**, respectively (Figure 2a,c). This aligns with studies showing that mutations at α1-GLN242 suppress neuroactive steroid PAM activity [32,33,34], highlighting the importance of this hydrogen bond in stabilizing ligand positioning and receptor activation. Moreover, the 4-cyanopyrazole group of Zuranolone and the 2-(trifluoroethoxy)pyridine-triazolone moiety of **S9** both form hydrogen bonds with β2-ARG312 (Figure 2). A 3D docking analysis indicated that the bond length of Zuranolone (2.6 Å) is slightly shorter than that of **S9** (3.2 Å) (Figure 2a,c), suggesting that Zuranolone has a slightly stronger interaction with the β2 subunit. In contrast, a 2D docking analysis demonstrated that **S9** exhibits a significantly shorter bond length (2.95 Å) compared to Zuranolone (3.9 Å) (Figure 2b,d), indicating a stronger binding affinity. Furthermore, while both **S9** and Zuranolone form extensive hydrophobic interactions with LEU301, TYR304, and ILE305 in the β2 subunit through their D rings, **S9** establishes an additional hydrophobic contact with ILE316 (Figure 2c,d). Both 2D and 3D docking analyses consistently showed that **S9** exhibits a larger hydrophobic interaction area compared to Zuranolone. The enhanced binding force and expanded hydrophobic network of **S9** likely contribute to the stabilization of the ligand–receptor complex, potentially improving its bioactive potency.

The electrophysiological data showed that the EC_50_ value of **S9** for synaptic α1β2γ2 receptors (50 ± 10 nmol/L) is significantly lower than that of Zuranolone (123 ± 20 nmol/L), indicating that **S9** has higher potency (Table 2). Therefore, it can be reasonably speculated that the new pharmacophore formed by the trifluoroethoxypyridine-triazolone substituent of **S9** may exhibit stronger binding to the β2 subunit and greater hydrophobic interactions, thereby enhancing its potency. In addition, the potency of **S9** for extrasynaptic α4β3δ receptors (EC_50:_ 34 ± 10 nmol/L) is also significantly higher than that of Zuranolone (93 ± 10 nmol/L) (Table 2). Since no cocrystal structure of the α4β3δ GABA_A_ receptor is currently available, whether the enhanced potency of **S9** for extrasynaptic receptors shares a mechanism similar to that for synaptic receptors remains to be confirmed by future studies.

### 2.5. In Vitro Studies of Bioactivity

#### 2.5.1. Study on GABA_A_ Receptor Pharmacology

Table 2 summarizes the pharmacological effects of Zuranolone and synthetic compounds **S1**–**S15** on GABA_A_ receptors in vitro. The data reveal that **S9** has a lower EC_50_ for both synaptic (α1β2γ2, 50 ± 10 nmol/L) and extrasynaptic (α4β3δ, 34 ± 10 nmol/L) GABA_A_ receptor subunits compared to Zuranolone (123 ± 20 nmol/L and 93 ± 10 nmol/L, respectively), indicating greater potency in modulating GABA_A_ receptor activity. However, **S9** exhibits a slight reduction in efficacy (E_max_%) for synaptic (675 ± 163%) and extrasynaptic (967 ± 118%) receptors compared to Zuranolone (894 ± 40% and 1368 ± 125%, respectively). In addition, the lower EC_50_ for extrasynaptic receptors (34 ± 10 nmol/L) compared to synaptic receptors (50 ± 10 nmol/L) suggests a more pronounced activation of δ-containing extrasynaptic GABA_A_ receptor subtypes. These results highlight that **S9** is more potent at lower concentrations and demonstrates a subtype-specific preference for extrasynaptic GABA_A_ receptors despite its slightly lower efficacy compared to Zuranolone.

#### 2.5.2. Study on Metabolic Stability in Liver Microsomes

The metabolic stability of Zuranolone and its analogs in rat (RLM) and human (HLM) liver microsomes was evaluated, as summarized in Table 3. In RLM, **S9** demonstrated significantly improved metabolic stability compared to Zuranolone, with a 6.5-fold lower intrinsic clearance (CL_int_ = 8 vs. 52 mL/min/mg) and a 4-fold longer half-life (T_1/2_ = 186 vs. 48 min). This metabolic advantage was also observed in HLM, where **S9** exhibited a 1.2-fold lower CL_int_ (9 vs. 11 mL/min/mg) and a 1.2-fold longer T_1/2_ (154 vs. 124 min) compared to Zuranolone. These results suggested that the structural modifications at C21 played a critical role in enhancing the metabolic profiles of the analog, with **S9** showing superior stability in both rat and human systems. In vitro human liver cytochrome (CYP) inhibition assays further demonstrated the metabolic stability of **S9**, with IC_50_ values greater than 30 μmol/L for multiple CYP isoenzymes (CYP1A2, 2C8, 2D6, and 3A4), reflecting a reduced risk of drug–drug interactions (Table 1).

### 2.6. In Vivo Evaluation of Bioactivities of Zuranolone and ***S9*** in Rats

#### 2.6.1. Pharmacokinetic Study of **S9** and Zuranolone

**Figure 3:**
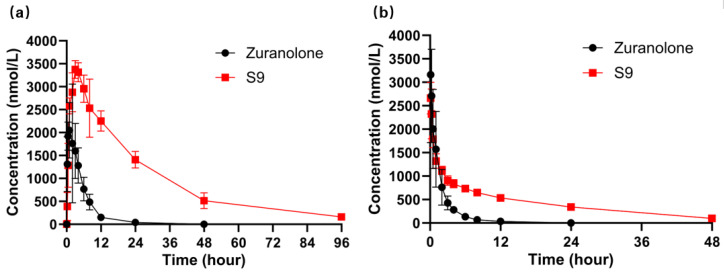
Concentration–time curves of **S9** and Zuranolone in rats after intragastric (**a**) (24.4 μmol/kg) and intravenous (**b**) (6.1 μmol/kg) administration (*n* = 6).

The pharmacokinetic study of **S9** and Zuranolone was carried out in rats following intragastric (i.g., 24.4 μmol/kg, *n* = 6) and intravenous (i.v., 6.1 μmol/kg, *n* = 6) administrations. The mean plasma concentration–time profiles for i.g. and i.v. administrations are presented in Figure 3a and Figure 3b, respectively, and the corresponding pharmacokinetic parameters are shown in Table 4.

After oral administration, **S9** in rats exhibited a rapid absorption phase, reaching a maximum plasma concentration (C_max_) of 3604 ± 541 nmol/L at approximately 3.7 h post-dosing. Subsequently, its concentration decreased over the next 24–96 h, indicating that the absorption of **S9** reached its maximum concentration within 4 h, and drug elimination became dominant in the subsequent test period. Zuranolone also had a rapid oral absorption phase but with a lower C_max_ of 2279 ± 554 nmol/L at around 1.2 h after administration, followed by a rapid decline in the next 6–24 h. The T_1/2_ of **S9** was 19.8 h, which was increased to approximately 5.2-fold that of Zuranolone at 3.8 h. The AUC_0–t_ of **S9** was 80,379 nmol/L·h, showing an increase of approximately 6.7-fold that of Zuranolone at 11,958 nmol/L·h. These results suggest that **S9** could markedly increase the oral absolute bioavailability compared to Zuranolone (82.9% vs. 57.7%). Following intravenous dosing, the T_1/2_ of **S9** was 16.4 h, which was increased to approximately 6.6-fold that of Zuranolone at 2.5 h. **S9** showed an increase in AUC_0–t_ to approximately 4.7-fold that of Zuranolone, at 24249 nmol/L·h compared to 5178 nmol/L·h.

Brain tissue analysis (Table 5) revealed distinct distribution patterns between the two compounds. Zuranolone showed rapid brain penetration with a high initial concentration (3103 nmol/kg at 0.5 h) but declined sharply to 193 nmol/kg by 6 h and fell below the quantification limit (BQL) at 24 h. In contrast, **S9** exhibited slower brain accumulation, with concentrations rising from 189 nmol/kg at 0.5 h to a peak of 2132 nmol/kg at 6 h and maintaining detectable levels (175 nmol/kg) even at 24 h. The total brain exposure (AUC_Brain_) of **S9** (30,969 nmol/kg·h) was increased to approximately 3.2-fold that of Zuranolone (9549 nmol/kg·h). Notably, **S9** displayed a relative brain bioavailability (F) of 324%, reflecting its superior ability to sustain therapeutic concentrations in the central nervous system.

#### 2.6.2. Effects of **S9** and Zuranolone on Pentylenetetrazole (PTZ)-Induced Acute Seizures

PTZ can trigger dose-dependent tonic seizures through its antagonistic effects on GABA_A_ receptor function. To evaluate target engagement at GABA_A_ receptors, Zuranolone and **S9** were tested in a PTZ-induced acute seizure model in rats, which is widely used to assess GABAergic activity. Based on their pharmacokinetic study results, **S9** or Zuranolone was administered orally at the same doses (6, 12, and 24 μmol/kg) 1 h prior to PTZ challenge.

**Figure 4:**
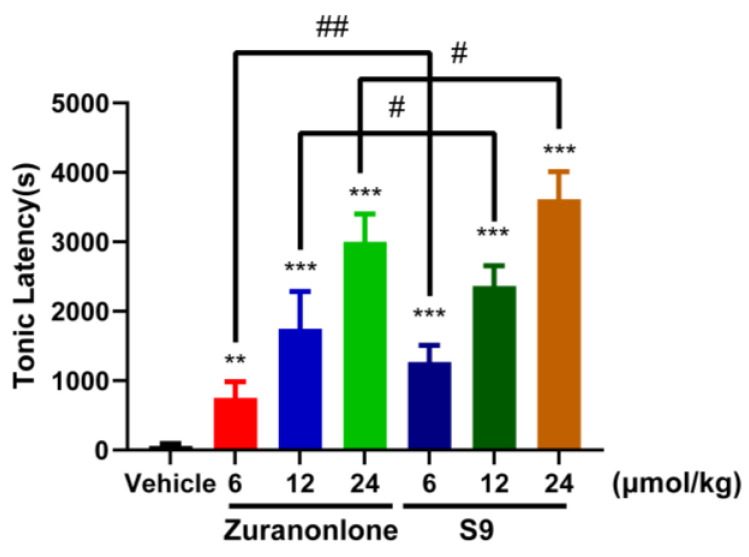
Intragastric administration of **S9** and Zuranolone attenuates acute seizure induced by PTZ in rats (*n* = 6). ** *p* < 0.01, *** *p* < 0.001 vs. vehicle treatment group; ^#^ *p* < 0.05, ^##^ *p* < 0.01 vs. Zuranolone treatment group.

Under the current experimental conditions, all the animal groups developed tonic seizures at different times, one after another, after the administration of PTZ. As shown in the results (Figure 4), oral administration of Zuranolone at 12 and 24 μmol/kg significantly prolonged the latency to tonic seizures (1748.0 ± 555.0 s and 2995.0 ± 430.6 s, respectively) compared to the vehicle group (61.7 ± 28.9 s; *p* < 0.001). Notably, **S9** demonstrated superior efficacy at equivalent molar doses, achieving seizure latency values of 2366.3 ± 304.1 s (12 μmol/kg) and 3613.2 ± 387.2 s (24 μmol/kg), with both doses showing statistically significant differences versus vehicle controls (*p* < 0.001). The effect of **S9** in prolonging the seizure latency is significantly stronger than that of Zuranolone (*p* < 0.01 for 6 μmol/kg; *p* < 0.05 for 12, 24 μmol/kg). Although both **S9** and Zuranolone prolonged the latency to tonic seizure onset, neither compound significantly affected seizure incidence. These findings confirm dose-dependent central pharmacodynamic effects of **S9** in the PTZ model and establish **S9**’s enhanced ability to suppress convulsive seizures relative to Zuranolone at matched concentrations.

#### 2.6.3. Effect of **S9** and Zuranolone on Loss of Righting Reflex (LORR)

An initial safety assessment of **S9** was conducted in response to central inhibition adverse effects observed with Zuranolone, including somnolence, confusion, and sedation [18]. We studied the anesthetic effects of intragastric administration of **S9** and Zuranolone by determining the threshold dose for LORR in rats, and the data were summarized in Table 6. **S9** significantly increased the LORR threshold dose (38.9 μmol/kg) to approximately 4-fold that of Zuranolone (9.8 μmol/kg). Furthermore, **S9**-treated groups exhibited reduced incidence of LORR and prolonged latency to first occurrence relative to Zuranolone, indicating an improved safety margin.

## 3. Discussion

The main aim of this study was to synthesize new analogs of Zuranolone by substituting the pyrazole group at the C21 position with triazolone derivatives with different electronic and structural properties to further overcome its therapeutic deficiencies. The results showed that the introduction of 2-(trifluoroethoxy)pyridine-triazolone (**S9**) onto Zuranolone at the C21 position, without increasing the lipophilicity, forms the unique pharmacophores that enhance the potency of GABA_A_ receptor activities and optimizes pharmacokinetic properties.

The preliminary study on SAR in this experiment mainly revealed that introducing pyridine derivatives containing different numbers of electronegative elements into the 2-triazolone substituent can exert impacts on the lipophilicity and biological activity of the compound molecules to varying extents. The results showed that the order of LogP values of 2-(trifluoroethoxy)pyridine (**S9**), 2-(difluoromethyl)pyridine (**S8**), 2-(fluoro)pyridine (**S6**), and pyridine (**S5**) (4.61 > 3.88 > 3.70 ≈ 3.78) shows a positive correlation with their potency and efficacy levels. NASs are highly lipophilic molecules with relatively high LogP values, and their high lipophilicity can influence potency and efficacy by preferentially accumulating in hydrophobic regions of cells, including the plasma membrane [27,28]. Based on this, not only the specific pharmacophore but also the nonspecific lipophilicity of NAS molecules is importantly considered for optimization of the potency and efficacy of NASs on GABA_A_ receptors [4,28,29]. Although the slight structural differences in the pyridine side chains of these compounds may have varying impacts on affinity, if this aspect is overlooked, it can be inferred that the introduction of multiple fluorine atoms is likely to greatly reduce the overall polarity and consequently increase lipophilicity and potency. Additionally, by using the ADMETlab 3.0 software applied in this study, we predicted that the LogP value of the previously reported 2,6-difluoropyridyl-triazolone derivative of **S28** is 4.028, which is lower than that of **S9** (4.61). According to the previously reported EC_50_ values for GABA_A_ receptors, the activity potency of **S28** is approximately 50% weaker than that of **S9**, which further validates the hypothesis regarding the positive correlation between lipophilicity and activity.

Furthermore, by comparing the lipophilicity between the highly active **S9** and Zuranolone, it was found that the LogP value of **S9** is slightly lower than that of Zuranolone (4.78), indicating its lesser lipophilicity. Nevertheless, the results showed that the potency of **S9** at GABA_A_ receptors is approximately 2.5-fold that of Zuranolone, whereas its efficacy is relatively lower, approximately 75%. Since the substantial difference in the potency between **S9** and Zuranolone cannot be explained by the slight variation in lipophilicity, it can be considered that the interactions between the nonspecific lipophilicity and the specific pharmacophore may be related to the overall functional performance. In particular, when the nonspecific lipophilicity of the molecules is approximately similar, the differences in potency may be mainly influenced by the properties of the pharmacophore [28,29]. To explore this, we conducted a comparative molecular docking study using the cocrystal structure of the synaptic α1β2γ2 subtype for **S9** and Zuranolone. The analysis results suggest that the trifluoroethoxypyridine-triazolone substituent of **S9** in the D-ring forms stronger hydrogen bonds and stronger hydrophobic interactions with the β2 subunit than those of the 4-cyanopyrazole group of Zuranolone. Therefore, we hypothesize that the trifluoroethoxypyridine-triazolone substituent’s unique structure, optimal hydrophobicity, and steric effects form a novel pharmacophore, potentially enhancing potency through increased receptor affinity. The exact mechanisms underlying the effect of nonspecific lipophilicity and specific pharmacophore of compound **S9** on its activity warrant further investigation.

Natural pregnane NASs exhibit a distinct characteristic in their preferential allosteric modulation of extrasynaptic GABA_A_ receptors. This is mainly because of GABA’s lower intrinsic activity and efficacy as a partial agonist at extrasynaptic δ-containing GABA_A_ receptors, thus the mildly increased sensitivity to low steroid concentrations [35,36,37]. The present data show that compound **S9**, similar to Zuranolone, displayed a greater potentiation of the potency at extrasynaptic GABA_A_ receptors to varying degrees than synaptic receptors, indicating that synthetic NAS drugs also have an inherent preference for acting on extrasynaptic receptors.

Moreover, **S9** can markedly enhance the potency of Zuranolone but causes a slight reduction in efficacy. It is generally believed that the reduced efficacy of **S9** on GABA_A_ receptors might weaken its therapeutic effects, particularly since the effects of NASs on seizure models are closely related to their efficacy [22,23]. PTZ-induced acute epilepsy is related to its blockade of GABA_A_ receptors and increased neuronal excitability. However, we observed that the anticonvulsant effect of **S9** on PTZ-induced acute epilepsy in rats is significantly more potent than that of Zuranolone. **S9** exhibits higher potency and bioavailability compared to Zuranolone, which may account for its more remarkable therapeutic efficacy. This result also confirms the function of **S9** in activating GABA_A_ receptors. On the other hand, the reduction in efficacy of **S9** on γ-containing synaptic GABA_A_ receptors might reduce its central inhibitory side effects [38], and we compared the anesthetic effects of **S9** and Zuranolone. The results show that the threshold dose of LORR in rats for **S9** is 4-fold that of Zuranolone, suggesting that **S9** has remarkably less central inhibition.

The new pharmacophore of **S9** has brought about very beneficial improvements to the biopharmaceutic properties. Predicted data show relatively low LogP and Log D values, along with a relatively high TPSA, suggesting better solubility. In addition, the weak inhibition of major human CYP isoenzymes indicates a lower risk of clinical drug–drug interactions. This modification of **S9** may improve metabolic stability (prolonged T_1/2_), reduce systemic clearance (higher AUC), and enhance blood–brain barrier permeability (increased AUC_Brain_). Compared to our previously reported Zuranolone analog (S28) [25], **S9** showed over 2-fold increases in T_1/2_ and AUC, along with over 1-fold enhanced GABA_A_ receptor potency, indicating an optimized structure and improved pharmacokinetics and bioactivity. However, more detailed metabolic and elimination pharmacokinetic studies on **S9** are necessary.

## 4. Materials and Methods

### 4.1. Materials

The main materials used in this experiment were sourced as follows: Zuranolone from BEIWANTABIO Co., Ltd. (Shanghai, China); liver microsomes (rat and human) and CYP isoenzymes (CYP1A2, CYP2C8, CYP2D6, CYP3A4) from XenoTech (Tewksbury, MA, USA); LTK cells from the China Center for Type Culture Collection (CCTCC, Wuhan, China); and CHO cells from the National Certified Cell Bank (Shanghai, China).

### 4.2. Animals

Male Sprague-Dawley rats (180–225 g) were sourced from Pengyue Laboratory Animal Technology Co., Ltd. (Jinan, China). All animal experiments were conducted under Specific Pathogen-Free (SPF) grade housing conditions.

### 4.3. Chemistry

#### 4.3.1. Structural Characterization

We purified the compounds using flash column chromatography and characterized them by spectroscopic analysis. Their purity (>95%) was confirmed using UHPLC-Q-Orbitrap mass spectrometry (MS) (SCIEX, Redwood City, CA, USA) and NMR spectroscopy (Bruker, Billerica, MA, USA). The structures of the 15 compounds were determined by ^1^H NMR, ^13^C NMR, and MS. All spectral data are provided in the Supplementary Materials.

#### 4.3.2. Synthesis

##### Synthesis of **S9**

Systematic modification of C21 substituents was conducted to improve target engagement profiles. As a representative example, the synthesis of **S9** (Scheme 1) adapted established Zuranolone methodology with key stereochemical modifications.

Catalytic hydrogenation of starting material **4** (Scheme 1) with 10 wt% Pd/C (H_2_, 1 atm, 25 °C, 16 h) yielded compound **5** as white crystals (mp 112–114 °C). Compound **5** was treated with MeMgBr (1.2 equiv, 0.5 M tetrahydrofuran solution) added dropwise to a solution (–78 °C) containing methylaluminum di-(2,6-di-tert-butyl-4-methylphenoxide) (MAD, 1.1 equiv) in dry toluene, then warmed to –20 °C over 4 h. TLC (hexane/EtOAc 4:1) confirmed complete conversion to compound **6** (dr > 99:1, ^1^H NMR), isolated in 41% yield via flash chromatography. The Wittig reaction of compound **6** with (ethoxycarbonylmethylene)triphenylphosphorane (1.5 equiv) in refluxing benzene (8 h) gave α, β-unsaturated ester **7** (88% yield, E/Z ratio 96:4). Hydroboration with 9-BBN (2.0 equiv, THF, 0 °C) followed by oxidative workup (3 M NaOH, 30% H_2_O_2_, 0 °C) produced alcohol **8** as a viscous liquid. Oxidation of alcohol **8** using pyridinium chlorochromate (PCC, 2.0 equiv, CH_2_Cl_2_, 4 Å molecular sieves, 2 h) cleanly converted it to compound **9** (91% yield). Bromination of compound **9** with PBr_3_ (**10**) (1.2 equiv, THF, 0 °C) followed by coupling with **11-9** (1.05 equiv, TBAB, K_2_CO_3_, DMF, 80 °C, 24 h) formed **S9** (76% overall yield, >99%). The detailed synthetic data of **S9** and relative intermediates can be confirmed in the Supplementary Materials.

##### Synthesis of **S1**–**S15**

For derivatives **S1**–**S14**, substituted hydrazines (**13-1**, **13-4**–**13-13**) were condensed with glyoxylic acid (1.2 equiv) in EtOH/H_2_O (1:1 *v*/*v*, pH 4.5, 60 °C) to give crystalline hydrazides (**14-1**, **14-4**–**14-13**), characterized by IR ν 1680 cm^−1^. Commercial **14-14** (Bide Pharmatech Ltd., Lot# BD-2305 Shanghai, China) was used as received. Treatment with diphenylphosphoryl azide (DPPA, 1.5 equiv, DMF, 0→25 °C) generated transient acyl azides, which underwent spontaneous cyclization (TEA, 80 °C, 12 h, Sigma-Aldrich, St. Louis, MO, USA) to form triazole derivatives (**11-1**, **11-4**–**11-13**) (Scheme 2). Tetrazole **11-2** was synthesized from methyl carbazate (**12**) and trimethoxymethane (1.5 equiv) in MeOH (40 °C, 4 h), followed by NaOMe-catalyzed cyclization with 4-fluoroaniline (**13**, 1.2 equiv) at 50 °C (72% yield, mp 145–147 °C). Similarly, **11-3** was obtained using cyclopropanemethylamine (**14**) in DMF under modified conditions (80 °C, 68% yield, Rf 0.33 in CHCl_3_/MeOH 9:1) (Scheme 2). The substituent of **S15**, 1-phenyl-1,3-dihydro-2H-benzo[d]imidazol-2-one (**11-15**), was commercially obtained. The detailed synthetic data of **S1**–**S8** and **S10**–**S15** can be confirmed in the Supplementary Materials.

### 4.4. Molecular Docking

Cocrystal structure of human GABA_A_ receptor α1β2γ2 subtype in complex with GABA plus AlloP was downloaded from the RCSB Protein Data Bank (https://www.rcsb.org/structure/8SI9, accessed on 26 December 2023). LigPrep in Maestro Schrödinger Suite 2018 (OPLS_2005 force field) generated the 3D models of Zuranolone and **S9**. Epik 2.2 in Schrödinger Suite generated ionization states at pH 7.0 ± 2.0, retaining 10 stereoisomers per ligand.

### 4.5. In Vitro and Vivo Studies of Bioactivity

#### 4.5.1. Quantification Analysis

We quantified **S1**–**S15** and Zuranolone using a UPLC-MS/MS (Agilent 1290 Infinity II UHPLC (Agilent Technologies, Santa Clara, CA, USA) coupled with a QTRAP 5500 MS/MS (ESI source) (SCIEX, Redwood City, CA, USA for MS/MS). The method assessed the content level of **S9** and Zuranolone in microsome metabolism and in rat brain tissue and plasma. An ACQUITY UPLC Peptide BEH C18 column (Waters; 50 × 2.1 mm, 1.7 μm; 60 °C; Waters Corporation, Milford, MA, USA) was used with a mobile phase of 0.02% propionic acid in water (A) and acetonitrile (B). The gradient elution of the mobile phase B is carried out as follows: it is 10% from 0 to 0.5 min, 90% from 0.8 to 2.5 min, and 10% from 2.8 to 3.5 min, with a flow rate of 0.40 mL/min. Quantitative analysis was performed on a QTRAP 5500 MS (SCIEX, Redwood City, CA, USA) in positive ionization mode with MRM (multiple reaction monitoring), using progesterone-d9 as the internal standard. MRM transitions and parameters were optimized for each analyte, with collision energies of 25 V (**S1**–**S15**), 23 V (Zuranolone), and 30 V (IS), and declustering potentials of 80 V (**S1**–**S15**), 90 V (Zuranolone), and 180 V (IS). Other conditions were as follows: curtain gas 10 psi, collision gas ‘medium’, ion spray voltage 5500 V, temperature 500 °C, and ionization source gases 1 and 2 at 50 psi.

#### 4.5.2. In Vitro Assay

##### Electrophysiology Assay

The effects of Zuranolone and synthetic analogs on GABA-evoked currents were assessed in LTK cells stably expressing α1β2γ2 GABA_A_ receptor subunits (*n* = 3) or CHO cells transiently expressing α4β3δ receptors (*n* = 3). Whole-cell mode recordings were conducted using the HEKA EPC-10 amplifier and analyzed with PatchMaster software (version 2x90, HEKA Elektronik, Lambrecht, Germany). High-throughput whole-cell recordings were performed using the Q-Patch method from Sophion. Test substances were dissolved in DMSO (dimethyl sulfoxide) to prepare 10 mmol/L stock solutions, then diluted with perfusion bath solution to concentrations ranging from 0.01 to 10 μmol/L. In this experiment, the effects of Zuranolone and its synthetic analogs on GABA-induced currents were examined in the presence of a submaximal agonist concentration at 1 μmol/L GABA (EC_20_). Cells were pre-incubated with increasing doses of test substances for 30 s, followed by 2 s of GABA stimulation, and currents were recorded. The maximum efficacy (Emax) of the GABA_A_ receptor is expressed with the current induced by 1 μmol/L GABA taken as 100%, and the percentage after treatment with the test compound is calculated. The EC_50_ values are determined by fitting the three concentration–response curves recorded from three cells.

##### Liver Microsome Assay

To evaluate the metabolic stability of compounds **S1**–**S15** and Zuranolone in RLM and HLM, we designed the following in vitro liver microsome metabolism assay protocol. In a 1.5 mL centrifuge tube, sequentially add 173 μL of PBS (0.1 M, pH 7.4), 5 μL of liver microsomes (20 mg/mL), and 2 μL of the test compound (100 μmol/L). After thorough mixing, pre-incubate at 37 °C for 10 min. Subsequently, add 20 μL of NADPH to initiate the metabolism, with a parallel control without NADPH, to validate the background signal. Samples were collected at 0, 5, 10, 20, 30, and 60 min post-reaction initiation. At each designated time point, we quenched the reaction with ice-cold acetonitrile (600 μL). After centrifuging the mixture, we analyzed the supernatant using UPLC-MS/MS. For data analysis, the compound concentration at 0 min was set as 100%, with concentrations at subsequent time points expressed as residual percentages relative to the initial value, and the in vitro half-life (T_1/2_) and intrinsic clearance (CL_int_) were calculated.

The IC_50_ values of **S9** and Zuranolone on CYP isoenzymes (1A2, 2C8, 2D6, 3A4) were determined in pooled HLM using a cocktail approach, as previously reported [39].

#### 4.5.3. In Vivo Evaluation in Rats

##### Pharmacokinetic Study

Zuranolone was solubilized in 5% DMSO with solutol HS 15 in saline. **S9** was solubilized in 10% DMSA with 30% SBECD (Zibo Qianhui Biological Technology Co., Ltd., Zibo, China) in saline. After fasting for 12 h, rats (*n* = 6 per group) were administered Zuranolone and **S9** intragastrically (i.g.) at a dose of 24.4 μmol/kg, and intravenously (i.v.) at 6.1 μmol/kg. Blood samples (200 μL) were collected into 1.5 mL tubes containing heparin at 15 min, 30 min, 1, 2, 3, 4, 6, 8, 12, 24, 48, and 96 h after i.g. or at 5, 15, 30 min, and 1, 2, 3, 4, 6, 8, 12, 24, 48, 96 h after i.v. Plasma was separated by centrifugation for 5 min (8000 rpm, 4 °C) and stored at −80 °C immediately.

##### Pentylenetetrazole (PTZ)-Induced Seizure Test

We randomly divided rats into 7 groups (*n* = 6): three same-dose groups for both Zuranolone and **S9** (6, 12, 24 μmol/kg) and a vehicle control group (30% SBECD). PTZ can selectively antagonize the GABA_A_ receptors at a dose below 60 mg/kg, while higher doses lead to non-competitive antagonism of GABA_A_ receptors and activation of NMDA receptors [40]. Thus, a dose of 50 mg/kg was applied in this experiment. After intragastric administration of the test drugs or vehicle for 1 h, the animals were injected intraperitoneally with PTZ saline solution at 50 mg/kg. The rats were then placed in observation chambers to monitor the latency of clonic seizures (seizures lasting ≥3 s with LORR) and tonic seizures (the rigid extension of limbs, forming an angle of more than 90° with the body plane) within 1 h.

##### Loss of Righting Reflex (LORR) Test

We randomly assigned rats into 6 groups (*n* = 10): Zuranolone groups at doses of 9.8, 15.6, and 19.5 μmol/kg and **S9** groups at doses of 38.9, 48.6, and 58.3 μmol/kg. The number of animals showing LORR, defined as the inability to turn over within 30 s while in the supine position, was recorded within 4 h following intragastric administration of the test drugs or vehicle.

### 4.6. Statistical Analysis

Experimental data were analyzed by GraphPad Prism software 9.0 and presented as means ± SD. Statistical differences were determined by one-way ANOVA followed by Dunnett’s post-hoc test, with *p* < 0.05 considered statistically significant. Main pharmacokinetic parameters were calculated using Phoenix WinNonlin 8.1 (non-compartmental model), and the absolute bioavailability of **S9** was determined by the following formula: (AUC i.g. (0–t) × dose i.v.)/(AUC i.v. (0–t) × dose i.g.) × 100%.

## 5. Conclusions

Our study attempted to develop novel GABA_A_ receptor-targeting therapeutics for addressing Zuranolone’s limitations and enhancing its efficacy and biopharmaceutic benefits. A rational balance of the influence of the lipophilicity of NAS drugs on the activity of GABA_A_ receptors and biopharmaceutic properties is one of the important considerations for optimizing steroidal backbone drugs. By introducing a trifluoroethoxypyridine-triazolone group (**S9**) onto the C21 position of Zuranolone, we observed enhanced GABA_A_ receptor potency and improved pharmacokinetic properties through optimized lipophilicity and pharmacophore interactions. The introduction of a substituent with a relatively large number of electronegative fluorine elements at the C21 position of **S9** demonstrates lower lipophilicity (lower LogP) and greater receptor binding affinity of the new pharmacophore, further enhancing the therapeutic advantages. **S9** exhibits higher potency towards the GABA_A_ receptor and higher bioavailability in vivo, showing stronger anticonvulsant efficacy against PTZ-induced acute seizures. Its slight low efficacy on synaptic GABA_A_ subunits remarkably increases the LORR threshold dose in rats, suggesting milder central inhibition. On the other hand, our results also demonstrate that the modest improvement in solubility and significantly enhanced metabolic stability of the **S9** molecule contribute to increased bioavailability, enhanced brain distribution, reduced clearance, and markedly prolonged plasma half-life. Thus, **S9**, without increased lipophilicity, exhibits favorable biopharmaceutic properties, increased activity potency, and enhanced safety, making it a highly promising candidate for the depression and/or epilepsy disorders and warranting further research.

## Figures and Tables

**Table 1:** Physicochemical parameters and inhibitions of CYP isozymes of Zuranolone and synthetic compounds.

Compounds	Zuranolone	S5	S6	S7	S8	S9	S13	S14	S15
pKa (acid) ^a^	10.8	9.52	9.07	10.4	9.68	10.3	10.0	10.5	12.0
LogP (pH 7.4) ^a^	4.78	3.79	3.70	4.53	3.88	4.61	4.20	4.52	5.75
LogD ^a^	4.39	3.75	3.57	4.24	3.78	4.13	3.95	4.16	4.91
TPSA (Å^2^) ^a^	78.9	90.0	90.0	90.0	90.0	99.2	100.9	86.4	64.2
CYP (IC_50_, μmol/L) ^b^: 1A2/2C8/2D6/3A4	>30	-	-	-	-	>30	-	-	-

^a^ Predicted data using ADMETlab3.0 software. ^b^ Experimental data using human liver cytochrome P450 (CYP) isoenzymes.

**Table 2:** The effects of Zuranolone and synthetic compounds on the potency (EC_50_) and maximum efficacy (E_max_%) of the GABA_A_ receptor subunits (*n* = 3).

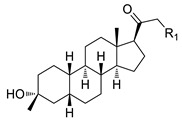					
Compounds	R_1_	α1β2γ2		α4β3δ	
		EC_50_ (nmol/L)	E_max_ (%)	EC_50_ (nmol/L)	E_max_ (%)
Zuranolone	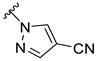	123 ± 20	894 ± 40	93 ± 10	1368 ± 125
**S1**	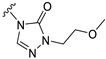	227 ± 52	735 ± 75	86 ± 25	609 ± 83
**S2**	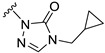	58 ± 10	320 ± 25	140 ± 10	450 ± 35
**S3**	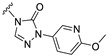	167 ± 37	389 ± 50	134 ± 32	474 ± 53
**S4**	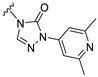	171 ± 40	691 ± 72	41 ± 19	563 ± 85
**S5**	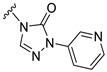	373 ± 45	773 ± 87	61 ± 15	391 ± 64
**S6**	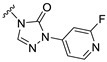	280 ± 52	736 ± 98	143 ± 30	507 ± 97
**S7**	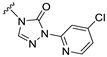	109 ± 27	624 ± 29	72 ± 21	589 ± 63
**S8**	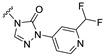	83 ± 24	600 ± 69	49 ± 15	432 ± 55
**S9**	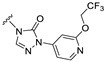	50 ± 10	675 ± 163	34 ± 10	967 ± 118
**S10**	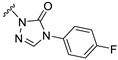	12.4 ± 5	294 ± 52	148 ± 25	428 ± 45
**S11**	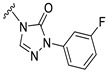	594 ± 51	495 ± 65	96 ± 23	936 ± 75
**S12**	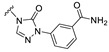	153 ± 33	764 ± 65	159 ± 20	386 ± 50
**S13**	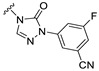	72 ± 16	518 ± 87	37 ± 12	549 ± 101
**S14**	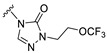	770 ± 104	576 ± 78	460 ± 59	889 ± 126
**S15**	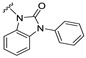	31 ± 12	305 ± 62	55 ± 15	470 ± 94

**Table 3:** The metabolic stability of Zuranolone and synthetic compounds in RLM and HLM.

Compounds	RLM		HLM	
	CL_int_ (mg/min/mg)	T_1/2_ (min)	CL_int_ (mg/min/mg)	T_1/2_ (min)
Zuranolone	52	48	11	124
**S1**	243	11	37	34
**S3**	161	16	17	73
**S5**	187	11	52	24
**S6**	83	34	60	40
**S7**	120	21	18	69
**S8**	108	29	53	23
**S9**	8	186	9	154
**S10**	219	10	38	33
**S12**	141	18	26	29
**S13**	27	90	25	40
**S14**	108	23	41	31
**S15**	7	210	5	248

RLM: Rat liver microsome; HLM: Human liver microsome.

**Table 4:** Pharmacokinetic parameters of **S9** and Zuranolone in rats (mean ± SD, *n* = 6).

Parameters	Zuranolone		S9	
	i.v.	i.g.	i.v.	i.g.
Dose (μmol/kg)	6.1	24.4	6.1	24.4
T_1/2_ (h)	2.5 ± 1.3	3.8 ± 1.7	16.4 ± 0.56	19.8 ± 1.5
T_max_ (h)	/	1.2 ± 0.8	/	3.7 ± 0.6
C_0_/C_max_ (nmol/L)	3432 ± 1737	2279 ± 554	2858 ± 223	3604 ± 541
AUC_0__–__t_ (nmol/L·h)	5178 ± 1732	11,958 ± 3057	24,249 ± 2325	80,379 ± 7942
F (%)	/	57.7%	/	82.9%

i.v.: Intravenous administration; i.g.: Intragastric administration.

**Table 5:** Brain distribution of **S9** and Zuranolone in rats (mean ± SD, *n* = 6).

Compounds ^a^	Brain (nmol/kg)				AUC_Brain 0–t_ (nmol/kg·h)	F_AUCBrain 0–t_ S9/Zuranolone (%)
	0.5 h	2 h	6 h	24 h		
Zuranolone	3103 ± 795	2220 ± 574	193 ± 56	BQL ^b^	9549 ± 2529	/
S9	189 ± 30	1895 ± 593	2132 ± 683	175 ± 35	30,969 ± 8450	324

^a^ Intragastric administration (24.4 μmol/kg); ^b^ Below the quantification limit.

**Table 6:** Effects of **S9** and Zuranolone on loss of righting reflex (LORR) in rats (*n* = 10).

Compounds	Dose ^a^ (μmol/kg)	Animals with LORR/Total (*n*)	Latency to LORR (min)
Zuranolone	19.5	9/10	30
	15.6	2/10	35
	9.8	0/10	/
S9	58.3	9/10	59
	48.6	6/10	68
	38.9	0/10	/

^a^ Intragastric administration.

## Data Availability

The original contributions presented in this study are included in the article/Supplementary Materials. Further inquiries can be directed to the corresponding author.

## Supplementary Materials

The following supporting information can be downloaded at: https://www.mdpi.com/article/10.3390/molecules30091918/s1, Method S1: Synthesis of compound **S9**; Method S2: Synthesis of compounds **S1**–**S8**, **S10**–**S15**; Figures S1–S6: The ^1^H-NMR spectra of the compounds **5**–**10**; Figures S7–S9: The ^1^H-NMR, ^13^C NMR and mass spectra of compound **S9**; Figures S10–S51: The ^1^H-NMR, ^13^C NMR and mass spectra of compounds **S1**–**S8** and **S10**–**S15**.
