# Supplementary material for: Synthesis and Evaluation of a Novel Zuranolone Analog with High GABAA Receptor PAM Activity and Excellent Pharmacokinetic Profiles
Source: Molecules. 2025 Apr 25;30(9):1918. doi: 10.3390/molecules30091918 (PMC12073189; doi:10.3390/molecules30091918)
Supplement: Supplementary file 1 [file molecules-30-01918-s001.zip › molecules-3581010-supplementary.pdf]

Supplemental Materials

Synthesis and Evaluation of a Novel Zuranolone Analog with High $GABA_A$ Receptor PAM Activity and Excellent Pharmacokinetic Profiles

Yingjie Yang, Xu Deng, Hengwei Xu, Daoyuan Chen, Fengjuan Zhao, Huijie Yang, Wenyan Wang, Chunjie Sha, Mingxu MA, Guanqing Zhang, Liang Ye, Jingwei Tian

Content	Page Number
Method 1: Synthesis of compound **S9**	P3−P4
Method 2: Synthesis of compounds **S1−S8**, **S10−S15**	P4−P7
Figure S1: ^{1}H NMR spectrum of compound **5**	P8
Figure S2: ^{1}H NMR spectrum of compound **6**	P8
Figure S3: ^{1}H NMR spectrum of compound **7**	P9
Figure S4: ^{1}H NMR spectrum of compound **8**	P9
Figure S5: ^{1}H NMR spectrum of compound **9**	P10
Figure S6: ^{1}H NMR spectrum of compound **10**	P10
Figure S7: ^{1}H spectrum of compound **S9**	P11
Figures S8, S9: ^{13}C NMR and Mass spectrum of compound **S9**	P12
Figures S10, S11: ^{1}H and ^{13}C NMR spectrum of compound **S1**	P13
Figure S12: Mass spectrum of compound **S1**	P14
Figures S13, S14: ^{1}H and ^{13}C NMR spectrum of compound **S2**	P15
Figure S15: Mass spectrum of compound **S2**	P16
Figures S16, S17: ^{1}H and ^{13}C NMR spectrum of compound **S3**	P17
Figure S18: Mass spectrum of compound **S3**	P18
Figures S19, S20: ^{1}H and ^{13}C NMR spectrum of compound **S4**	P19
Figure S21: Mass spectrum of compound **S4**	P20
Figures S22, S23: ^{1}H and ^{13}C NMR spectrum of compound **S5**	P21
Figure S24: Mass spectrum of compound **S5**	P22
Figures S25, S26: ^{1}H and ^{13}C NMR spectrum of compound **S6**	P23
Figure S27: Mass spectrum of compound **S6**	P24
Figures S28, S29: ^{1}H and ^{13}C NMR spectrum of compound **S7**	P25
Figure S30: Mass spectrum of compound **S7**	P26
Figures S31, S32: ^{1}H and ^{13}C NMR spectrum of compound **S8**	P27
Figure S33: Mass spectrum of compound **S8**	P28
Figures S34, S35: ^{1}H and ^{13}C NMR spectrum of compound **S10**	P29
Figure S36: Mass spectrum of compound **S10**	P30
Figures S37, S38: ^{1}H and ^{13}C NMR spectrum of compound **S11**	P31
Figure S39: Mass spectrum of compound **S11**	P32
Figures S40, S41: ^{1}H and ^{13}C NMR spectrum of compound **S12**	P33
Figure S42: Mass spectrum of compound **S12**	P34
Figures S43, S44: ^{1}H and ^{13}C NMR spectrum of compound **S13**	P35
Figure S45: Mass spectrum of compound **S13**	P36
Figures S46, S47: ^{1}H and ^{13}C NMR spectrum of compound S14	P37
Figure S48: Mass spectrum of compound **S14**	P38
Figures S49, S50: ^{1}H and ^{13}C NMR spectrum of compound **S15**	P39
Figure S51: Mass spectrum of compound **S15**	P40

Method 1: Synthesis of compound **S9**

*(5R,8R,9R,10S,13S,14S)-13-methyltetradecahydro-3H-cyclopenta[a]phenanthrene-3,17(2H)-dione (**5**).*

Starting material 4 was obtained commercially. A mixture of material **4** (200 g, 734 mM, 1.0 *eq*) and HBr (5.96 g, 35.4 mM, 0.048 *eq*) in tetrahydrofuran (THF, 1400 mL) was treated with 10 wt% Pd/C (10.0 g, 9.43 mM, 0.013 *eq*). The reaction system was maintained under a hydrogen atmosphere (1.0 MPa) at 25 °C for 24 h with continuous agitation. Reaction progress was monitored by TLC (dichloromethane/methanol = 50:1), confirming complete consumption of **4** (Rf = 0.30 for product). The crude mixture was filtered through Celite, and the filtrate was concentrated under reduced pressure. The residue was triturated in acetone (600 mL), filtered, and dried to yield intermediate 5 (180 g, crude) as a white solid. ^{1}H NMR (400 MHz, $CDCl_3$): δ 2.65–2.38 (m, 2H), 2.29–2.05 (m, 6H), 2.03–1.93 (m, 1H), 1.90–1.70 (m, 3H), 1.69–1.59 (m, 3H), 1.58–1.49 (m, 2H), 1.46–1.11 (m, 6H), 0.91 (s, 3H).

*(3R,5R,8R,9R,10S,13S,14S)-3-Hydroxy-3,13-dimethylhexadecahydro-17H-cyclopenta[a]phenanthren-17-one (**6**).*

To a cooled (−60 °C) solution of **5** (100 g, 364 mM, 1.0 *eq*) in toluene (1.0 L), bis(2,6-di-tert-butyl-4-methylphenoxy)methylalumane (525 g, 1.09 mol, 3.0 *eq*) was added. After 1 h of stirring at −60 °C, methylmagnesium bromide (3 M in THF, 364 mL, 3.0 *eq*) was introduced dropwise. The reaction was stirred for an additional 3 h at −60 °C, with TLC (dichloromethane/methanol = 20:1) indicating partial conversion (Rf = 0.21 for product). The mixture was quenched with saturated NH_4Cl (500 mL), extracted with ethyl acetate (2 × 200 mL), and the combined organic layers were washed with brine (100 mL), dried (Na_2SO_4), and concentrated. Silica gel chromatography (dichloromethane/methanol = 100:1 to 10:1) formed 6 (46 g, 44%) as a white solid. ^{1}H NMR (400 MHz, $CDCl_3$): δ 2.43 (dd, J = 8.4, 19.2 Hz, 1H), 2.13–2.01 (m, 1H), 1.98–0.97 (m, 25H), 0.86 (s, 3H).

*(3R,5R,8R,9R,10S,13S,14S,E)-17-ethylidene-3,13-dimethylhexadecahydro-1H-cyclopenta[a]phenanthren-3-ol (**7**).*

Ethyltriphenylphosphonium bromide (102 g, 275 mM, 4.0 *eq*) and potassium tert-butoxide (30.9 g, 275 mM, 4.0 *eq*) were combined in THF (200 mL) at 0 °C. After 1 h of heating at 60 °C, a solution of 6 (20.0 g, 68.9 mM, 1.0 *eq*) in THF (120 mL) was added. The reaction proceeded at 60 °C for 13 h, with TLC (petroleum ether/ethyl acetate = 3:1) confirming full conversion (Rf = 0.50). Workup with saturated NH_4Cl (150 mL), extraction (EtOAc, 2 × 150 mL), and chromatography (petroleum ether/EtOAc = 100:1→3:1) yielded 7 (18.3 g, 88%) as a colorless solid. ^{1}H NMR (400 MHz, $CDCl_3$): δ 5.11 (td, J = 2.0, 7.2 Hz, 1H), 2.48–2.30 (m, 1H), 2.27–2.08 (m, 2H), 1.95–1.78 (m, 3H), 1.74–0.98 (m, 24H), 0.88 (s, 3H).

*(3R,5R,8R,9R,10S,13S,14S,17S)-17-((R)-1-Hydroxyethyl)-3,13-dimethylhexadecahydro-1H-cyclopenta[a]phenanthren-3-ol (**8**).*

A solution of **7** (18.3 g, 60.6 mM, 1.0 *eq*) in THF (180 mL) was treated with $BH_3 \cdot Me_2S$ (10.0 M, 30.3 mL, 5.0 *eq*) at 0 °C. After 3 h at 25 °C, NaOH (3 M, 100 mL) and H_2O_2 (86.5 g, 763 mM, 12.6 *eq*) were added dropwise. The mixture was stirred for 4 hours at 25 °C, quenched with saturated Na_2SO_3 (2 × 60 mL), extracted with EtOAc (2 × 150 mL), and purified by chromatography (petroleum ether/EtOAc = 100:1→3:1) to form 8 (13 g, 67%) as a white solid. ^{1}H NMR (400 MHz, $CDCl_3$): δ 3.85–3.56 (m, 1H), 1.94–1.73 (m, 6H), 1.70–1.52 (m, 6H), 1.51–1.15 (m, 20H), 1.05–0.90 (m, 3H).

*1-((3R,5R,8R,9R,10S,13S,14S,17S)-3-hydroxy-3,13-dimethylhexadecahydro-1H-cyclopenta[a]phenanthren-17-yl) ethan-1-one (**9**).*

Pyridinium chlorochromate (PCC, 17.5 g, 81.1 mM, 2.0 eq) was added to a solution of 8 (13.0 g, 40.6 mM, 1.0 eq) in dichloromethane (130 mL) at 0 °C. After 3 h at 25 °C, the reaction was

quenched with saturated Na_2SO_3 (100 mL), extracted with CH_2Cl_2 (2 × 100 mL), and chromatographed (petroleum ether/EtOAc = 100:1→1:1) to yield 9 (9 g, 70%) as a white solid. ^{1}H NMR (400 MHz, $CDCl_3$): δ 2.53 (t, J = 8.8 Hz, 1H), 2.11 (s, 3H), 1.81 – 1.23 (m, 26H), 0.61 (s, 3H).

*2-bromo-1-((3R,5R,8R,9R,10S,13S,14S,17S)-3-hydroxy-3,13-dimethylhexadecahydro-1H-cyclopenta[a]phenanthren-17-yl) ethan-1-one (**10**).*

Bromine (4.53 g, 28.3 mM, 1.0 *eq*) and HBr (927 mg, 5.50 mM, 0.19 *eq*) were added to a solution of 9 (9.03 g, 28.3 mM, 1.0 *eq*) in methanol (90 mL) at 0 °C. After 12 h at 25 °C, the mixture was neutralized with saturated $NaHCO_3$ (50 mL), extracted with EtOAc (2 × 50 mL), and chromatographed (petroleum ether/EtOAc = 100:1→1:1) to form 10 (7.1 g, 63%) as a white solid. ^{1}H NMR (400 MHz, $CDCl_3$): δ 3.99–3.85 (m, 2H), 2.92–2.69 (m, 1H), 2.25–2.12 (m, 1H), 1.98–1.58 (m, 9H), 1.53–1.28 (m, 11H), 1.27–1.18 (m, 3H), 1.15–1.03 (m, 3H), 0.64 (s, 3H).

*4-(2-((3R,5R,8R,9R,10S,13S,14S,17S)-3-hydroxy-3, 13-dimethylhexadecahydro-1H-cyclopenta[a]phenanthren-17-yl)-2-oxoethyl)-2-(2-(2,2,2-trifluoroethoxy)pyridin-4-yl)-2,4-dihydro-3H-1,2,4-triazol-3-one (**S9**).*

A solution of **10** (100 mg, 384 μM, 1.1 *eq*) and 7 (139 mg, 349 μM, 1.0 *eq*) in THF (10 mL) was treated with K_2CO_3 (483 mg, 3.49 mM, 10.0 *eq*) under nitrogen. The reaction was heated at 50 °C for 16 h, quenched with water, and extracted with EtOAc (3 × 20 mL). Chromatographic purification (petroleum ether/EtOAc = 10:1) yielded S9 (136 mg, 63.1%) as a white solid. ^{1}H NMR (600 MHz, DMSO-d_6): δ 8.26–8.24 (m, 2H), 7.64-7.63 (dd, J = 5.8, 1.8 Hz, 1H), 7.41 (d, J = 1.8 Hz, 1H), 5.06-4.99 (q, J = 9.0 Hz, 2H), 4.75-4.55 (d, J = 18.5 Hz, 2H), 4.25 (d, J = 18.5 Hz, 1H), 2.79-2.76 (s, 1H),2.09-2.07(m,, 2H) ,1.76-1.67 (m, J = 8.9 Hz, 8H), 1.35-1.28 (m, J = 7.7 Hz, 9H), 1.25–1.10 (m, 7H), 0.58 (s, 3H). ^{13}C NMR (151 MHz, DMSO-d_6): δ 203.41, 162.30, 151.17, 148.09, 146.72, 140.27, 107.05, 96.82, 69.72, 59.40, 55.20, 51.74, 44.58, 41.18, 41.03, 39.66, 39.38, 39.24, 37.87, 37.16, 34.31, 33.99, 31.09, 26.28, 25.81, 25.24, 24.96, 23.92, 22.44, 13.09. HRMS (ESI): m/z calcd for C30H40F3N4O4 $[M+H]^+$ 577.29962; found 577.29944.

Method 2: Synthesis of compounds **S1–S8, S10–S15**

*4-(2-((3R,5R,8R,9R,10S,13S,14S,17S)-3-hydroxy-3,13-dimethylhexadecahydro-1H-cyclopenta[a]phenanthren-17-yl)-2-oxoethyl)-2-(pyridin-3-yl)-2,4-dihydro-3H-1,2,4-triazol-3-one (**S1**).*

The product **S1** was prepared by a method similar to that for synthesizing **S9**. Yield: 105 mg (63.8%), obtained as a white solid. ^{1}H NMR (400 MHz, DMSO-d_6): δ = 7.82 (s, 1H), 4.65-4.39 (m, 2H), 3.83-3.75 (m, 2H), 3.58-3.55 (m, 2H), 3.21 (s, 3H), 2.74-2.70 (m, 1H), 2.08-1.99 (m, 2H), 1.72-1.06 (m, 25H), 0.56 (s, 3H). ^{13}C NMR (151 MHz, DMSO-d_6): δ 203.84, 152.65, 137.16, 69.70, 68.90, 59.39, 57.78, 55.18, 51.49, 44.45, 44.17, 41.16, 41.01, 37.85, 37.15, 34.30, 33.97, 31.08, 26.27, 25.80, 25.23, 24.95, 23.91, 22.41, 20.13, 13.07. HRMS (ESI): m/z calcd for C26H42N3O4 $[M+H]^+$ 460.31698; found 460.31693.

*4-(cyclopropylmethyl)-2-(2-((3R,5R,8R,9R,10S,13S,14S,17S)-3-hydroxy-3,13-dimethylhexadecahydro-1H-cyclopenta[a]phenanthren-17-yl)-2-oxoethyl)-2,4-dihydro-3H-1,2,4-triazol-3-one (**S2**).*

The product **S2** was prepared in a similar manner to the synthesis of **S9**. Yield: 80 mg (27.1%), as a white solid. MS (ESI) m/z =455.3 [M+1]+, ^{1}H NMR (400 MHz, DMSO-d_6): δ 8.02 (s, 1H), 4.76-4.57 (m, 1H), 4.55-4.37 (m, 1H), 3.43 (br d, J = 7.2 Hz, 2H), 2.68 (br s, 1H), 2.22-2.09(m, 2H), 1.82 – 1.08 (m, 26H), 0.61-0.44 (m, 5H), 0.38-0.28 (m, 2H). ^{13}C NMR (151 MHz, DMSO-d6): δ 204.21, 153.18, 136.70, 69.70, 59.31, 55.23, 54.89, 46.01, 44.43, 41.15, 41.01, 339.92, 38.06, 37.16, 34.29, 33.97, 31.07, 26.25, 25.80, 25.22, 24.95, 23.92, 22.35, 13.17, 10.57, 3.34, 3.30. HRMS (ESI): m/z calcd for C27H42N3O3 $[M+H]^+$ 456.32207; found 456.32150.

*4-(2-((3R,5R,8R,9R,10S,13S,14S,17S)-3-hydroxy-3,13-dimethylhexadecahydro-1H-cyclopenta[a]phenanthren-17-yl)-2-oxoethyl)-2-(6-methoxypyridin-3-yl)-2,4-dihydro-3H-1,2,4-triazol-3-one (**S3**).*

The product **S3** was prepared by a method similar to that for the synthesis of **S9**. Yield: 100 mg (31.1%), obtained as a white solid. ^{1}H NMR (400 MHz, DMSO-*d*$_6$): δ = 8.61 (d, J = 2.4 Hz, 1H), 8.15-8.12 (m, 2H), 6.95 (d, J = 8.8 Hz, 1H), 4.72-4.54 (m, 2H), 3.87 (s, 3H), 2.76 (s, 2H), 2.10-2.05 (m, 2H), 1.73-1.67 (m, 7H), 1.38-1.32 (m, 9H), 1.28-1.06 (m, 8H), 0.59 (s, 3H). ^{13}C NMR (151 MHz, DMSO-*d*$_6$): δ 203.64, 160.95, 151.00, 139.13, 136.72, 130.10, 128.93, 110.69, 69.71, 59.41, 55.19, 53.44, 51.72, 44.53, 41.16, 41.02, 40.04, 37.86, 37.15, 34.31, 33.97, 31.07, 26.28, 25.80, 25.23, 24.95, 23.92, 22.42, 13.10. HRMS (ESI): m/z calcd for C29H41N4O4 [M+H]$^+$ 509.31223; found 509.31198.

*2-(2,6-dimethylpyridin-4-yl)-4-(2-((3R,5R,8R,9R,10S,13S,14S,17S)-3-hydroxy-3,13-dimethylhexadecahydro-1H-cyclopenta[a]phenanthren-17-yl)-2-oxoethyl)-2,4-dihydro-3H-1,2,4-triazol-3-one (**S4**).*

Product **S4** was prepared using a method similar to that for the synthesis of **S9**. Yield: 65.4 mg (35.1%), obtained as a white solid. ^{1}H NMR (400 MHz, DMSO-*d*$_6$): δ = 8.44 (s, 1H), 8.09 (s, 2H), 4.80-4.58 (m, 2H), 2.80-2.78 (br t, J = 8.8 Hz, 1H), 2.72-2.70 (s, 6H), 2.07-2.04 (br d, J = 9.6 Hz, 2H), 1.72-1.03 (m, 24H), 0.58 (s, 3H). ^{13}C NMR (151 MHz, DMSO-*d*$_6$): δ 203.70, 155.09, 151.85, 142.51, 110.73, 70.19, 59.78, 55.67, 52.47, 45.14, 41.65, 41.49, 38.33, 37.64, 34.79, 34.45, 31.56, 26.77, 26.28, 25.72, 25.44, 24.41, 22.93, 19.96, 13.63. HRMS (ESI): m/z calcd for C30H43N4O3 [M+H]$^+$ 507.33297; found 507.33328.

*4-(2-((3R,5R,8R,9R,10S,13S,14S,17S)-3-hydroxy-3,13-dimethylhexadecahydro-1H-cyclopenta[a]phenanthren-17-yl)-2-oxoethyl)-2-(pyridin-3-yl)-2,4-dihydro-3H-1,2,4-triazol-3-one(**S5**).*

The product **S5** was prepared following a method similar to that for the synthesis of **S9**. Yield: 70 mg (22.8%), obtained as a white solid. ^{1}H NMR (400 MHz, DMSO-*d*$_6$): δ 9.11 (d, J=2.12 Hz, 1H), 8.45-8.48 (m, 1H), 8.22-8.27 (m, 2H), 7.52 (dd, J=8.38, 4.63 Hz, 1H), 4.69-4.76 (m, 1H), 4.54-4.62 (m, 1H), 4.25 (s, 1H), 2.77 (bt, J=8.82 Hz, 1H), 2.02-2.12 (m, 2H), 1.56-1.78 (m, 7H), 1.13-1.39 (m, 10H), 0.97-1.12 (m, 7H), 0.59 (s, 3H). ^{13}C NMR (151 MHz): δ 203.56, 151.14, 146.19, 139.76, 139.06, 134.22, 124.85, 123.97, 69.71, 59.40, 55.19, 51.74, 44.55, 41.16, 41.01, 39.08, 37.85, 37.15, 34.31, 33.97, 31.07, 26.28, 25.80, 25.23, 24.95, 23.92, 22.43, 13.11.HRMS (ESI): m/z calcd for C28H39N4O3 [M+H]$^+$ 479.30167; found 479.30170.

*2-(2-fluoropyridin-4-yl)-4-(2-((3R,5S,8R,9S,10S,13S,14S,17S)-3-hydroxy-3,10,13-trimethylhexadecahydro-1H-cyclopenta[a]phenanthren-17-yl)-2-oxoethyl)-2,4-dihydro-3H-1,2,4-triazol-3-one (**S6**).*

Product **S6** was prepared by a method similar to that for the synthesis of **S9**. Yield: 110 mg (62.16%), obtained as a white solid. ^{1}H NMR (400 MHz, $CDCl_3$): δ = 8.23 (d, J = 5.8 Hz, 1H), 7.88 (d, J = 5.6 Hz, 1H), 7.71-7.53 (m, 2H), 4.69-4.33 (m, 2H), 2.63 (t, J = 8.8 Hz, 1H), 2.33-2.15 (m, 1H), 2.11 (br d, J = 11.4 Hz, 1H), 1.81-1.63 (m, 4H), 1.55-1.39 (m, 6H), 1.37-1.13 (m, 12H), 1.05-0.95 (m, 1H), 0.83 (br d, J = 2.1 Hz, 1H), 0.77 (s, 3H), 0.68 (s, 3H). ^{13}C NMR (151 MHz, DMSO-*d*$_6$): δ 203.38, 164.70, 163.16, 151.12, 148.90, 147.94, 140.63, 109.68, 96.00, 67.52, 59.16, 56.10, 53.63, 51.77, 44.44, 41.62, 40.17, 37.81, 35.13, 35.08, 34.47, 33.60, 31.75, 28.05, 24.05, 22.39, 20.52, 13.14, 11.10. HRMS (ESI): m/z calcd for C29H38FN4O3 [M-H]-509.29334; found 509.29306.

*2-(4-chloropyridin-2-yl)-4-(2-((3R,5R,8R,9R,10S,13S,14S,17S)-3-hydroxy-3,13-dimethylhexadecahydro-1H-cyclopenta[a]phenanthren-17-yl)-2-oxoethyl)-2,4-dihydro-3H-1,2,4-triazol-3-one (**S7**).*

Product **S7** was prepared by a method similar to that for the synthesis of **S9**. Yield: 70 mg (32.8%), a white solid. ^{1}H NMR (400 MHz, DMSO-*d*$_6$): δ = 8.48 (d, J = 5.6 Hz, 1H), 8.17-8.16 (m, 1H), 8.02 (d, J = 1.6 Hz, 1H), 7.46 (dd, J = 2.0, 5.4 Hz, 1H), 4.73-4.54 (m, 2H), 4.42-4.37 (m, 1H), 2.79-2.73 (m, 1H), 2.11-2.04 (m, 2H), 1.79-1.64 (m, 7H), 1.41-1.23 (m, 9H), 1.11 (s, 8H), 0.58 (s,

3H). ^{13}C NMR (151 MHz, DMSO-d6): δ 203.53, 151.25, 150.21, 149.99, 144.30, 139.68, 121.46, 69.71, 59.37, 55.19, 51.71, 44.57, 41.16, 41.01, 39.91, 39.08, 37.85, 37.15, 34.31, 33.97, 31.07, 26.27, 25.80, 25.23, 24.95, 23.92, 22.43, 13.10. HRMS (ESI): m/z calcd for C28H38ClN4O3 $[M+H]^+$ 513.26270; found 513.26306.

*2-(2-(difluoromethyl)pyridin-4-yl)-4-(2-((3R,5R,8R,9R,10S,13S,14S,17S)-3-hydroxy-3,13-dimethylhexadecahydro-1H-cyclopenta[a]phenanthren-17-yl)-2-oxoethyl)-2, 4-dihydro-3H-1,2,4-triazol-3-one (**S8**).*

Product **S8** was prepared using a method similar to that for the synthesis of **S9**. Yield: 26 mg (34.78%), a white solid. ^{1}H NMR (400 MHz, DMSO-d_6): δ = 8.66 (d, J = 5.6 Hz, 1H), 8.30 (d, J = 2.0 Hz, 1H), 8.10 (d, 1H), 7.67 (s, 1H), 6.66 (s, H), 4.76-4.28 (m, 2H), 2.73-2.54 (m, 1H), 2.14 (s, 2H), 1.89-1.62 (m, 9H), 1.54-1.20 (m, 16H), 0.69 (s, 3H). ^{13}C NMR (151 MHz, DMSO-d_6): δ 203.38, 153.34, 151.23, 144.85, 140.61, 115.05, 113.47, 112.63, 111.88, 107.51, 69.71, 59.36, 55.19, 51.79, 44.59, 41.16, 41.01, 37.85, 37.15, 34.31, 33.97, 31.07, 26.28, 25.80, 25.23, 24.95, 23.92, 22.43, 13.12. HRMS (ESI): m/z calcd for C29H39F2N4O3 $[M+H]^+$ 529.29847; found 529.29852.

*4-(4-fluorophenyl)-2-(2-((3R,5R,8R,9R,10S,13S,14S,17S)-3-hydroxy-3,13-dimethylhexadecahydro-1H-cyclopenta[a]phenanthren-17-yl)-2-oxoethyl)-2,4-dihydro-3H-1,2,4-triazol-3-one (**S10**).*

The product **S10** was prepared using a method similar to that for the synthesis of **S9**. Yield: 200 mg (47.9%), a white solid. ^{1}H NMR (400 MHz, DMSO-d_6): δ 8.49 (s, 1H), 7.71-7.77 (m, 2H), 7.38 (t, J =8.88 Hz, 2H), 4.73-4.81 (m, 1H), 4.58 (d, J =18.4 Hz, 1H), 4.22-4.27 (m, 1H), 2.75 (s, 1H), 2.00-2.08 (m, 3H), 1.57-1.77 (m, 8H), 1.28-1.41 (m, 6H), 0.93-1.12 (m, 9H), 0.58 (s, 3H). ^{13}C NMR (151 MHz, DMSO-d_6): δ 204.01, 161.32, 159.70, 151.70, 135.26, 130.37, 123.65, 116.29, 69.71, 62.88, 59.74, 59.35, 55.23, 55.10, 44.50, 41.17, 41.01, 38.09, 37.16, 34.30, 33.97, 31.08, 26.26, 25.81, 25.23, 24.95, 23.93, 22.36, 13.18. HRMS (ESI): m/z calcd for C29H39FN3O3 $[M+H]^+$ 496.29700; found 496.29691.

*2-(3-fluorophenyl)-4-(2-((3R,5S,8R,9S,10S,13S,14S,17S)-3-hydroxy-3,10,13-trimethylhexadecahydro-1H-cyclopenta[a]phenanthren-17-yl)-2-oxoethyl)-2,4-dihydro-3H-1,2,4-triazol-3-one (**S11**).*

The product **S11** was prepared using a method similar to that for the synthesis of **S9**. Yield: 20 mg (16.02%), obtained as a white solid. ^{1}H NMR (400 MHz, DMSO-d_6): δ = 8.21 (s, 1H), 7.84-7.69 (m, 2H), 7.64-7.43 (m, 1H), 7.11 (br d, J = 2.0 Hz, 1H), 4.81-4.55 (m, 2H), 3.88 (s, 1H), 2.85-2.67 (m, 1H), 2.22-1.90 (m, 2H), 1.80 -0.75 (m, 23H), 0.74 (s, 3H), 0.60 (s, 3H). ^{13}C NMR (151 MHz, DMSO-d_6): δ 203.61, 163.03, 161.42, 150.97, 139.20, 131.15, 113.29, 111.67, 104.61, 104.25, 67.53, 59.18, 56.11, 53.63, 51.69, 44.42, 41.62, 40.18, 37.81, 35.14, 35.09, 34.47, 33.60, 31.76, 31.70, 28.06, 24.05, 22.39, 20.53, 13.14. HRMS (ESI): m/z calcd for C29H39FN3O3 $[M+H]^+$ 496.29700; found 496.29736.

*3-(4-(2-((3R,5R,8R,9R,10S,13S,14S,17S)-3-hydroxy-3,13-dimethylhexadecahydro-1H-cyclopenta[a]phenanthren-17-yl)-2-oxoethyl)-5-oxo-4,5-dihydro-1H-1,2,4-triazol-1-yl)benzamide (**S12**).*

The product **S12** was prepared using a method similar to that employed for the synthesis of **S9**. Yield: 135 mg (58.8%), obtained as a white solid. ^{1}H NMR (400 MHz, DMSO-d_6): δ = 8.41-8.35 (m, 1H), 8.19 (s, 1H), 8.09-8.03 (m, 1H), 8.11-8.03 (m, 1H), 7.77-7.70 (m, 1H), 7.55 (t, J = 8.0 Hz, 1H), 7.46 (br s, 1H), 4.76-4.55 (m, 2H), 4.26 (s, 1H), 2.82-2.72 (m, 1H), 2.16-2.03 (m, 2H), 1.80-1.62 (m, 7H), 1.35 (br d, J = 9.6 Hz, 7H), 1.30-1.16 (m, 4H), 1.12 (s, 4H), 1.06 (br d, J = 10.8 Hz, 2H), 0.60 (s, 3H). ^{13}C NMR (151 MHz, DMSO-d_6): δ 203.65, 167.37, 151.05, 138.98, 137.68, 135.45, 129.06, 123.91, 120.25, 117.18, 69.72, 59.74, 59.40, 55.19, 51.71, 44.58, 41.17, 41.02, 37.86, 37.15, 34.31, 33.98, 31.08, 26.28, 25.80, 25.24, 24.96, 23.93, 22.43, 13.11. HRMS (ESI): m/z calcd for C30H39N4O4 [M-H]-519.29768; found 519.29779.

*3-fluoro-5-(4-(2-((3R,5R,8R,9R,10S,13S,14S,17S)-3-hydroxy-3,13-dimethylhexadecahydro-1H-cyclopenta[a]phenanthren-17-yl)-2-oxoethyl)-5-oxo-4, 5-dihydro-1H-1,2,4-triazol-1-yl)benzonitrile (**S13**).*

Product **S13** was prepared by a method similar to that for the synthesis of **S9**. Yield: 144 mg (60.7%), obtained as a white solid. ^{1}H NMR (400 MHz, DMSO-d_6): δ = 8.28 (s, 1H), 8.16 (s, 1H), 8.05 (td, J = 2.4, 10.5 Hz, 1H), 7.80-7.74 (m, 1H), 4.83-4.47 (m, 2H), 4.25 (br s, 1H), 2.77 (br t, J = 8.8 Hz, 1H), 2.12-2.01 (m, 2H), 1.78-1.01 (m, 24H), 0.59 (s, 3H). ^{13}C NMR (151 MHz, DMSO-d_6): δ 203.37, 162.79, 161.16, 151.00, 140.01, 117.22, 116.66, 115.74, 115.57, 113.54, 109.39, 109.21, 69.71, 59.36, 55.18, 51.78, 44.57, 41.16, 37.85, 37.15, 34.30, 33.97, 31.07, 26.27, 25.80, 25.23, 24.95, 23.91, 22.42, 13.10. HRMS (ESI): m/z calcd for C30H36FN4O3 [M-H]-519.27769; found 519.27771.

*4-(2-((3R,5R,8R,9R,10S,13S,14S,17S)-3-hydroxy-3,13-dimethylhexadecahydro-1H-cyclopenta[a]phenanthren-17-yl)-2-oxoethyl)-2-(2-(trifluoromethoxy)ethyl)-2,4-dihydro-3H-1,2,4-triazol-3-one (**S14**).*

Product **S14** was prepared using a method similar to that for synthesizing **S9**. Yield: 160 mg (60.9%), obtained as a white solid. ^{1}H NMR (400 MHz, $CDCl_3$): δ = 7.46 (s, 1H), 4.55-4.33 (dd, 2H), 4.27-4.25 (d, 2H), 4.14-4.09 (m, 2H), 2.58-2.63 (m, 1H), 2.32-1.09 (m, 27H), 0.67 (s, 3H). ^{13}C NMR (151 MHz, DMSO-d_6): δ 203.71, 152.72, 137.75, 122.04, 120.35, 69.70, 65.30, 59.38, 55.18, 51.53, 44.46, 43.66, 41.16, 41.01, 37.84, 37.15, 34.30, 33.97, 31.07, 26.26, 25.80, 25.22, 24.95, 23.91, 22.40, 13.06. HRMS (ESI): m/z calcd for C26H37F3N3O4 [M-H]-512.27416; found 512.27423.

*1-(2-((3R,5R,8R,9R,10S,13S,14S,17S)-3-hydroxy-3,13-dimethylhexadecahydro-1H-cyclopenta[a]phenanthren-17-yl)-2-oxoethyl)-3-phenyl-1, 3-dihydro-2H-benzo[d]imidazol-2-one (**S15**).*

Product **S15** was prepared using a method similar to that for the synthesis of **S9**. Yield: 88 mg (34.8%), obtained as a pale yellow solid. 1H NMR (400 MHz, DMSO-d_6): δ 7.71-7.39 (m, 5H), 7.22-6.98 (m, 4H), 5.06-4.61 (m, 2H), 4.24 (s, 1H), 2.23-2.04 (m, 3H), 1.74-1.11 (m, 24H), 0.61 (s, 3H). ^{13}C NMR (151 MHz, DMSO-d_6): δ 204.21, 152.54, 134.34, 129.64, 129.50, 128.67, 127.52, 125.72, 121.87, 121.38, 108.51, 108.29, 69.71, 59.48, 55.28, 50.84, 44.49, 41.18, 41.01, 38.14, 37.17, 34.30, 33.98, 31.08, 26.28, 25.81, 25.31, 24.96, 23.96, 22.51, 20.75, 14.07, 13.14. HRMS (ESI): m/z calcd for C34H43N2O3 $[M+H]^+$ 527.32682; found 527.32697.

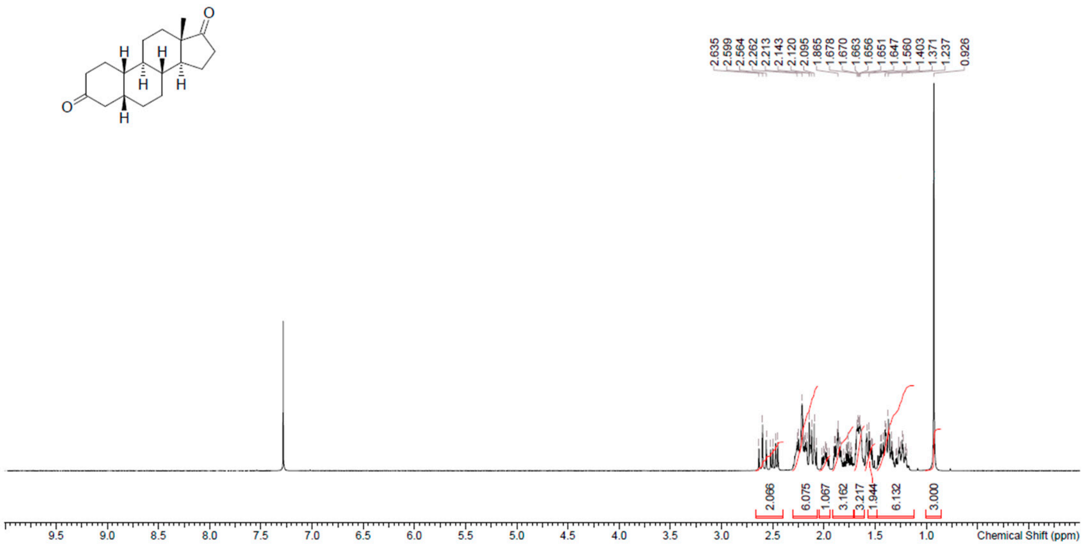

Figure S1. ^{1}H NMR (400 MHz, $CDCl_3$) spectrum of compound **5**:

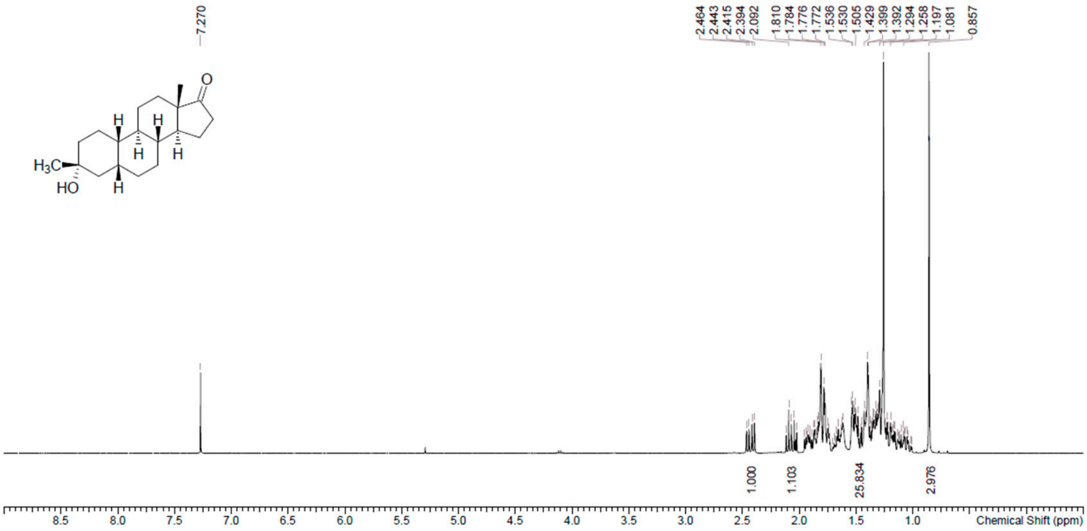

Figure S2. ^{1}H NMR (400 MHz, $CDCl_3$) spectrum of compound **6**

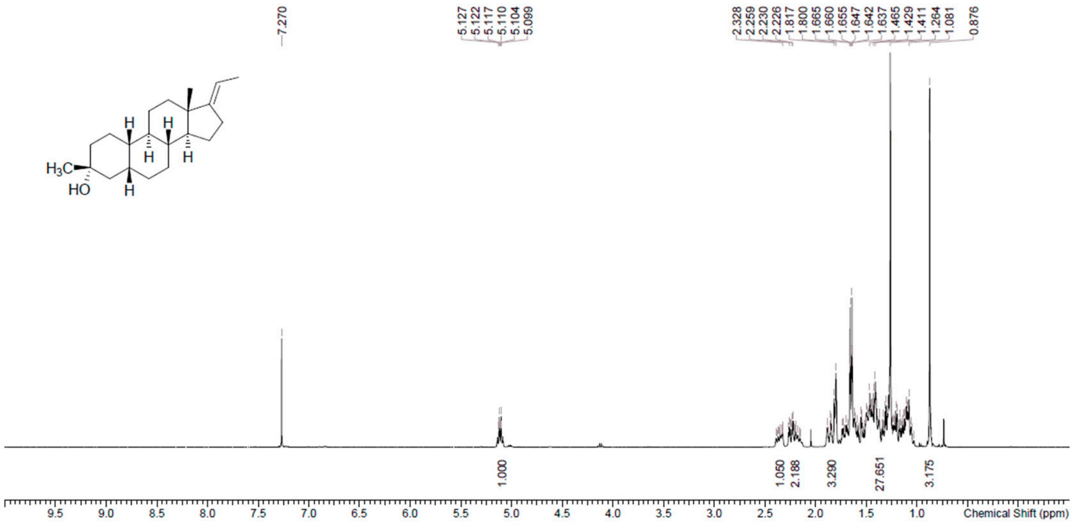

Figure S3. ^{1}H NMR (400 MHz, $CDCl_3$) spectrum of compound **7**.

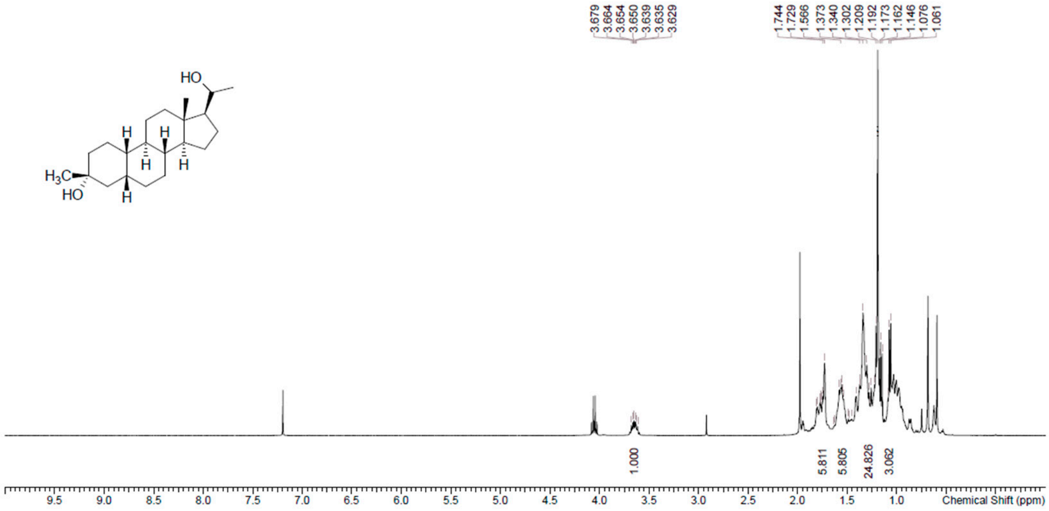

Figure S4. ^{1}H NMR (400 MHz, $CDCl_3$) spectrum of compound **8**

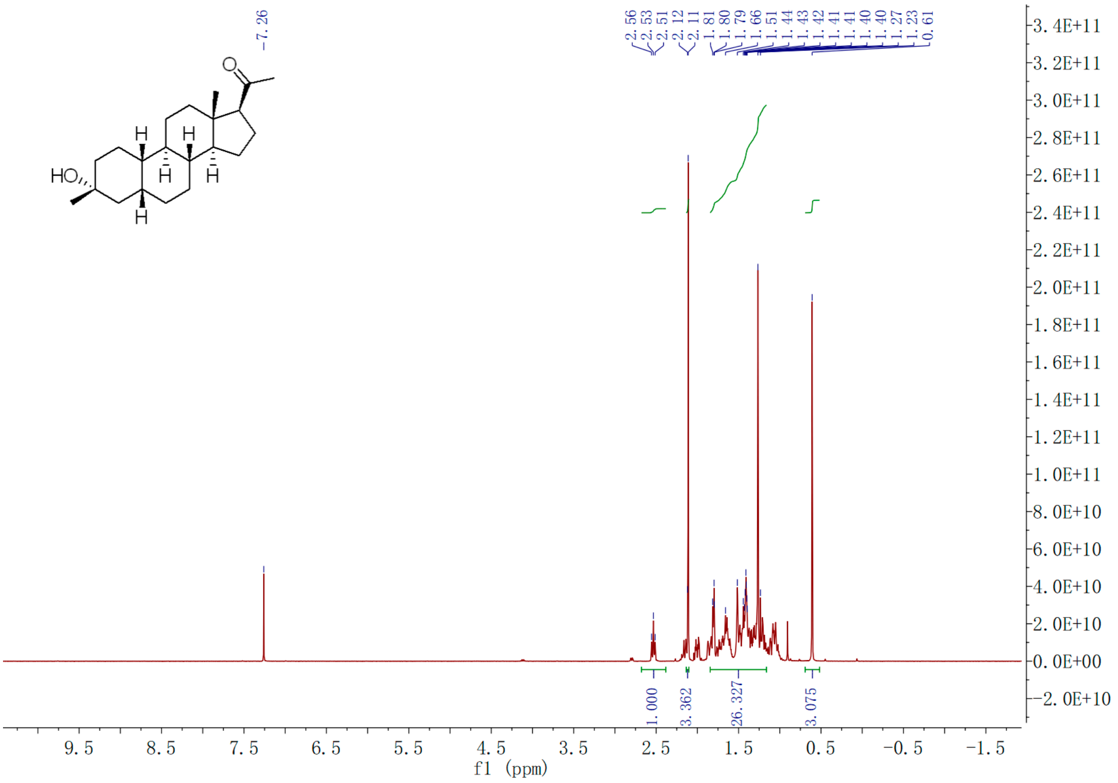

Figure S5. ^{1}H NMR (400 MHz, $CDCl_3$) spectrum of compound **9**

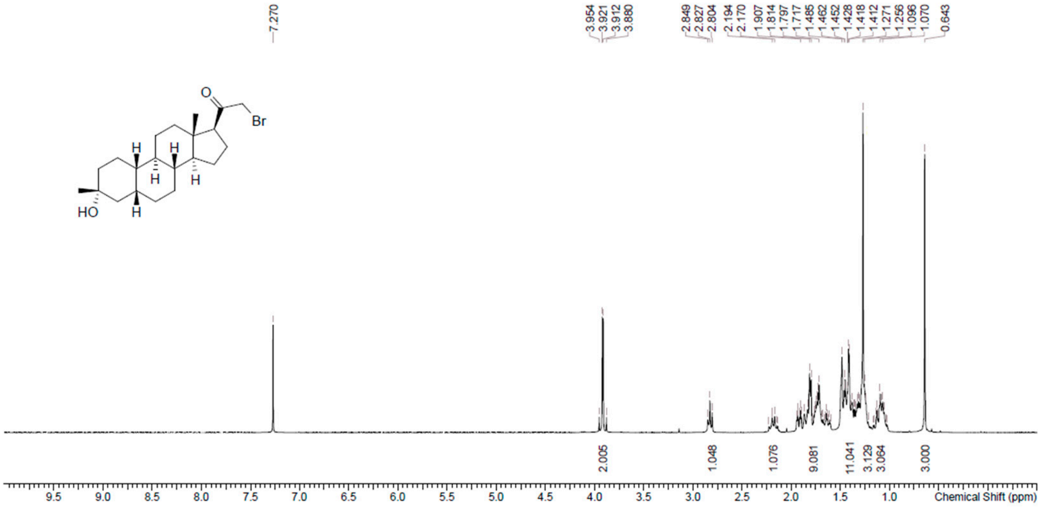

Figure S6. ^{1}H NMR (400 MHz, $CDCl_3$) spectrum of compound **10**

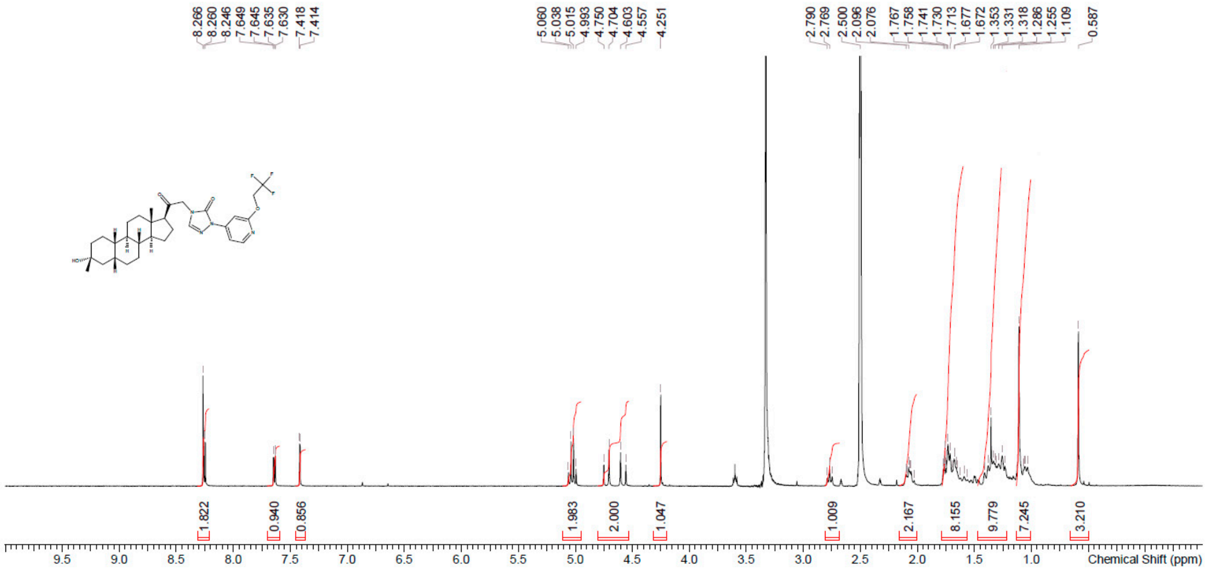

Figure S7. ^{1}H NMR (600 MHz, DMSO-d_6) spectrum of compound **S9**

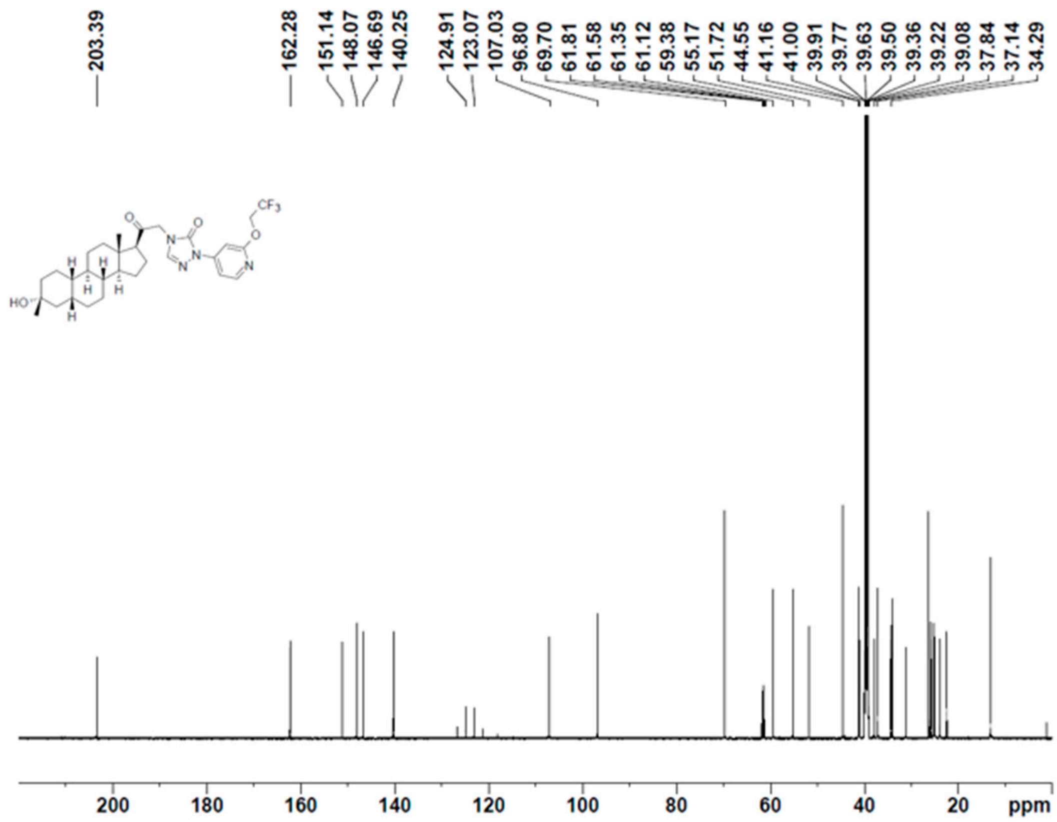

Figure S8. ^{13}C NMR (151 MHz, DMSO-d_6) spectrum of compound **S9**

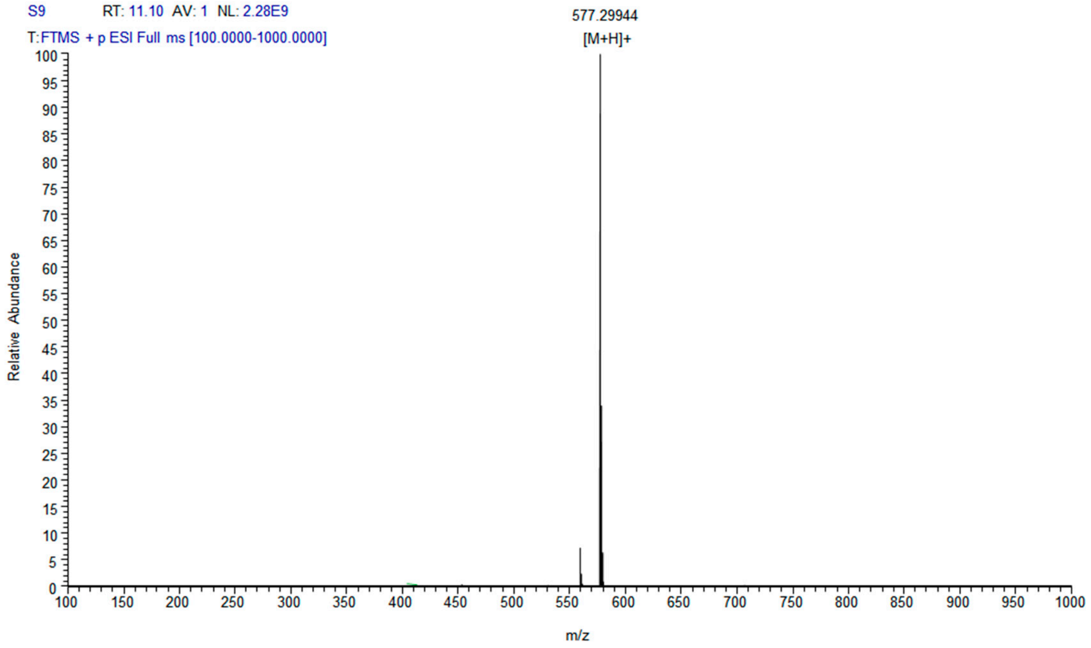

Figure S9. HRMS (ESI): m/z calcd for $C_{30}H_{40}F_3N_4O_4$ $[M+H]^+$ 577.29962; found 577.29944.

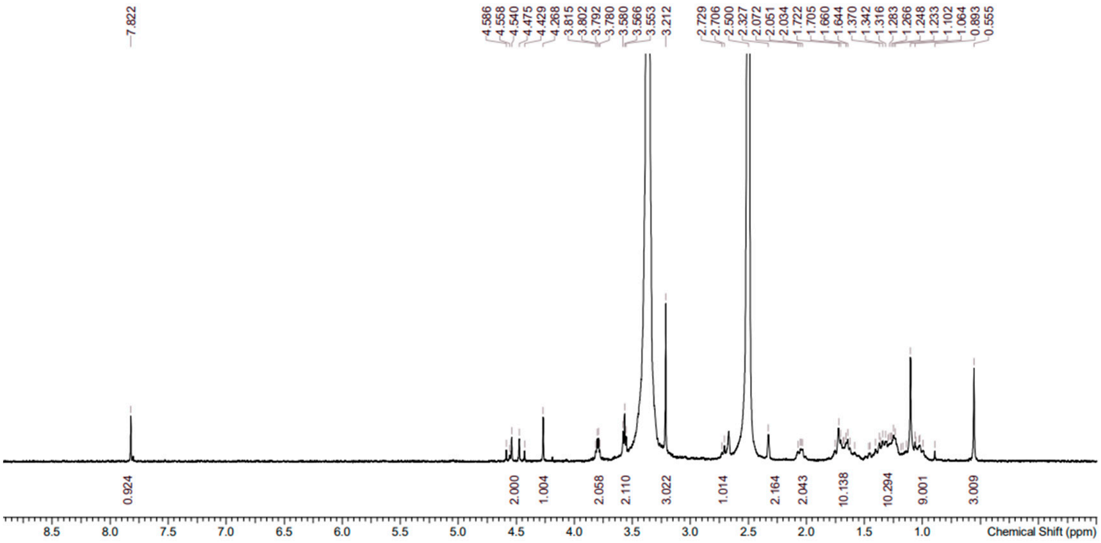

Figure S10. ^{1}H NMR (400 MHz, DMSO-d_6) spectrum of compound **S1**

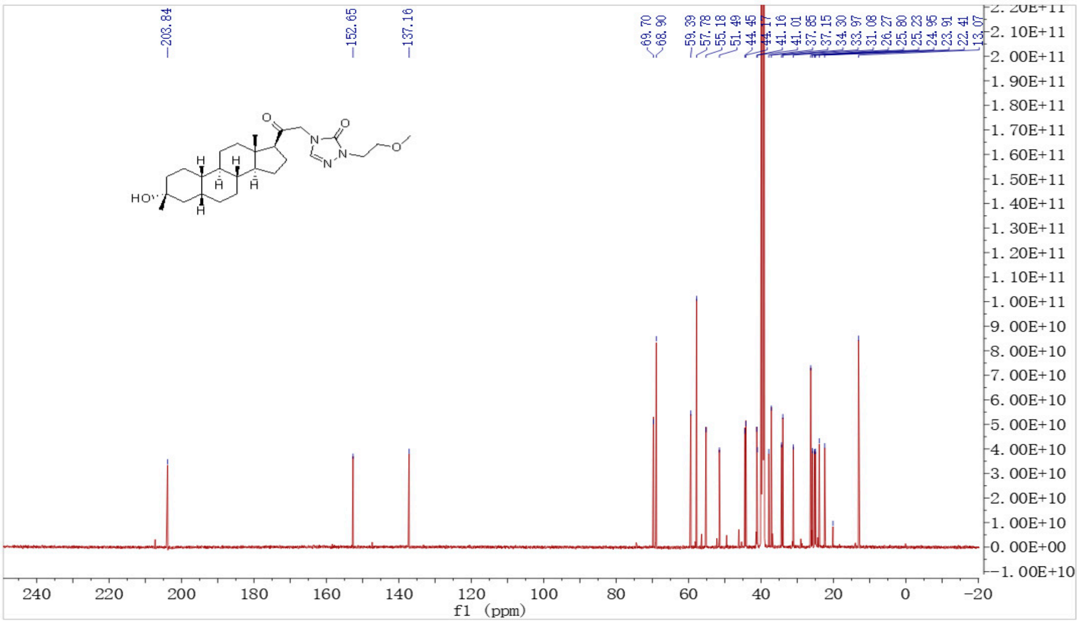

Figure S11. ^{13}C NMR (151 MHz, DMSO-d_6) spectrum of compound **S1**

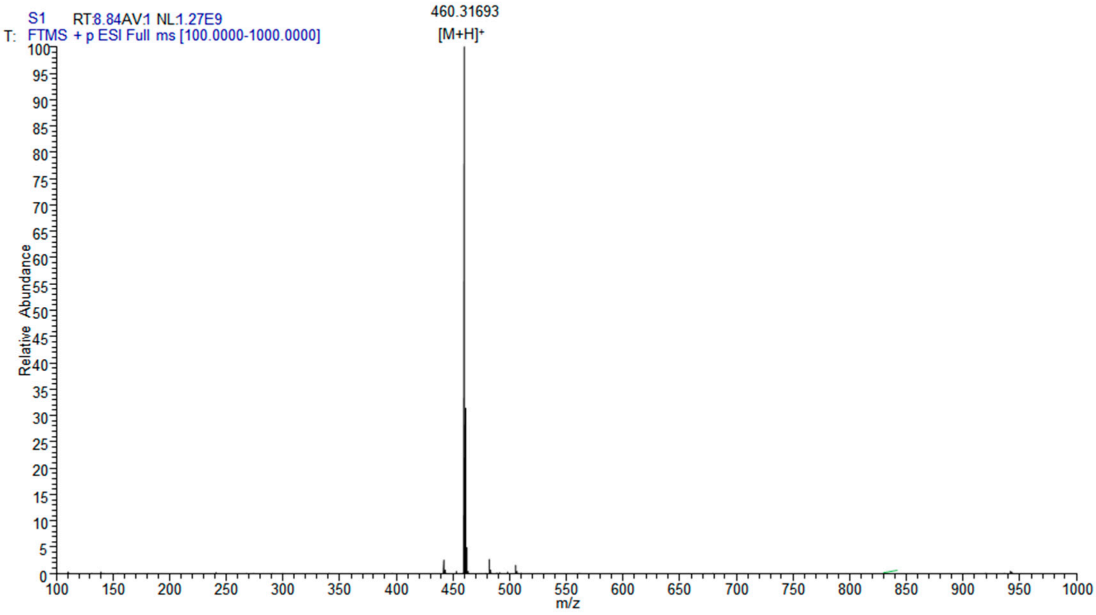

Figure S12. HRMS (ESI): m/z calcd for $C_{26}H_{42}N_3O_4$ $[M+H]^+$ 460.31698; found 460.31693.

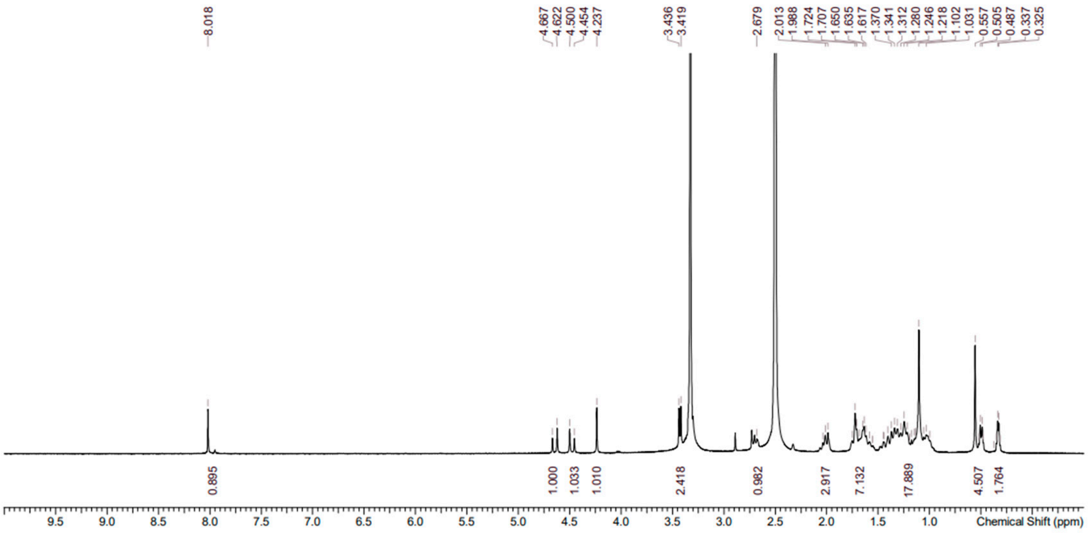

Figure S13. ^{1}H NMR (400 MHz, DMSO-d_6) spectrum of compound **S2**

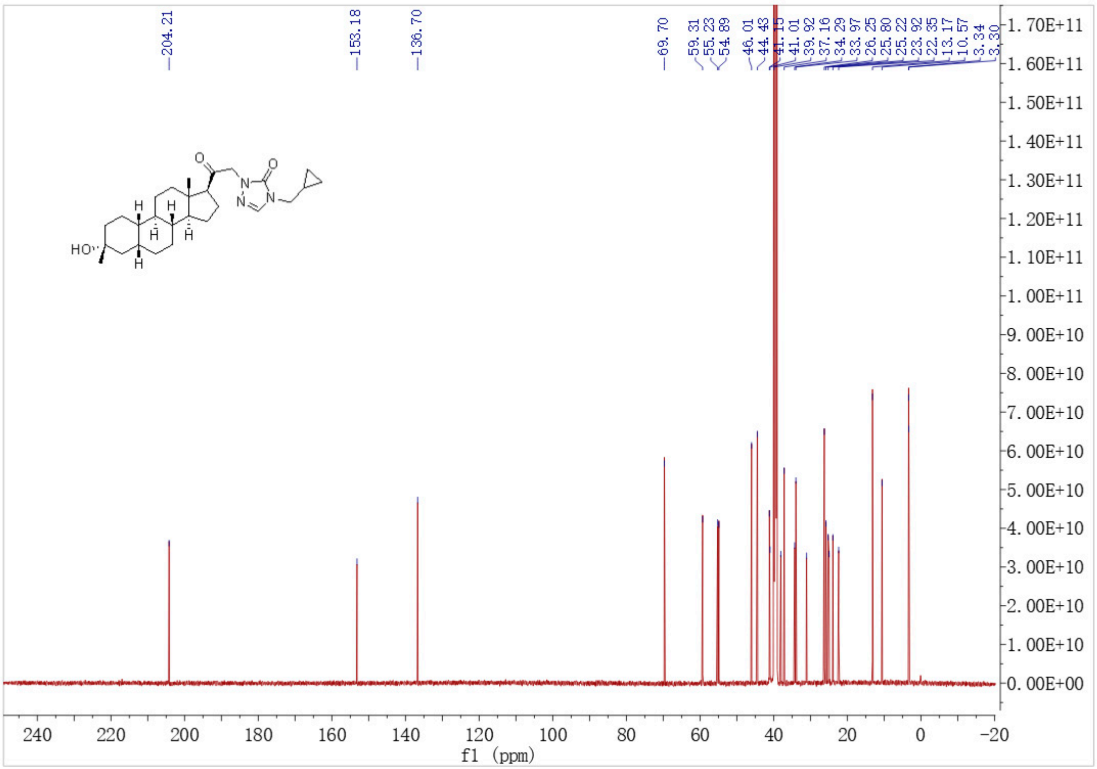

Figure S14. ^{13}C NMR (151 MHz, DMSO-d_6) spectrum of compound **S2**

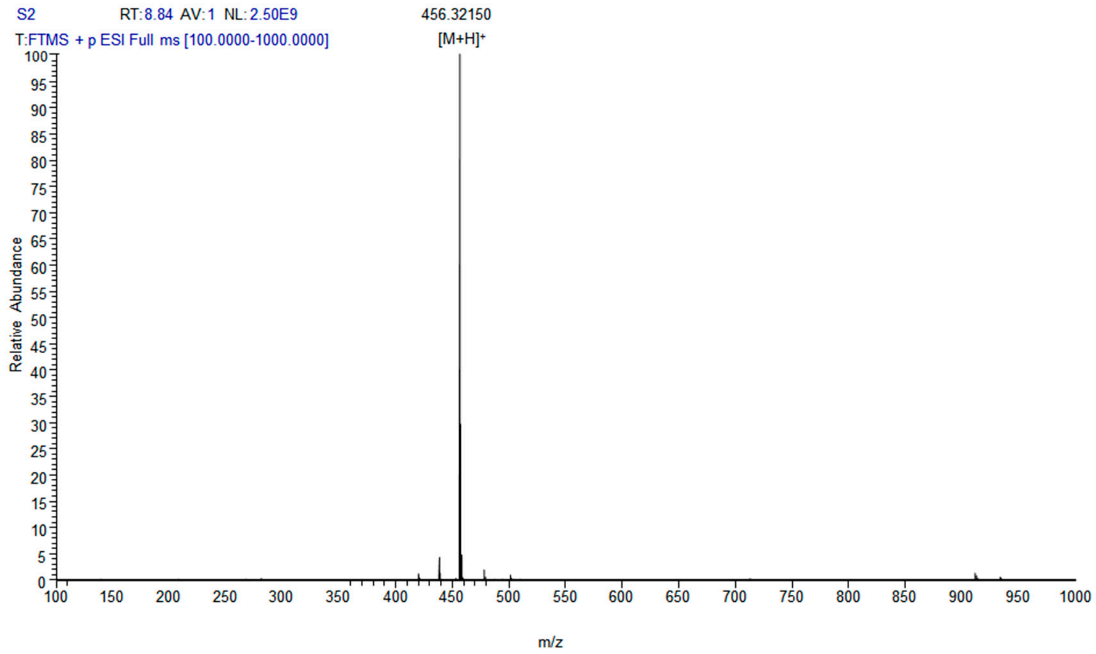

Figure S15. HRMS (ESI): m/z calcd for C27H42N3O3 $[M+H]^+$ 456.32207; found 456.32150.

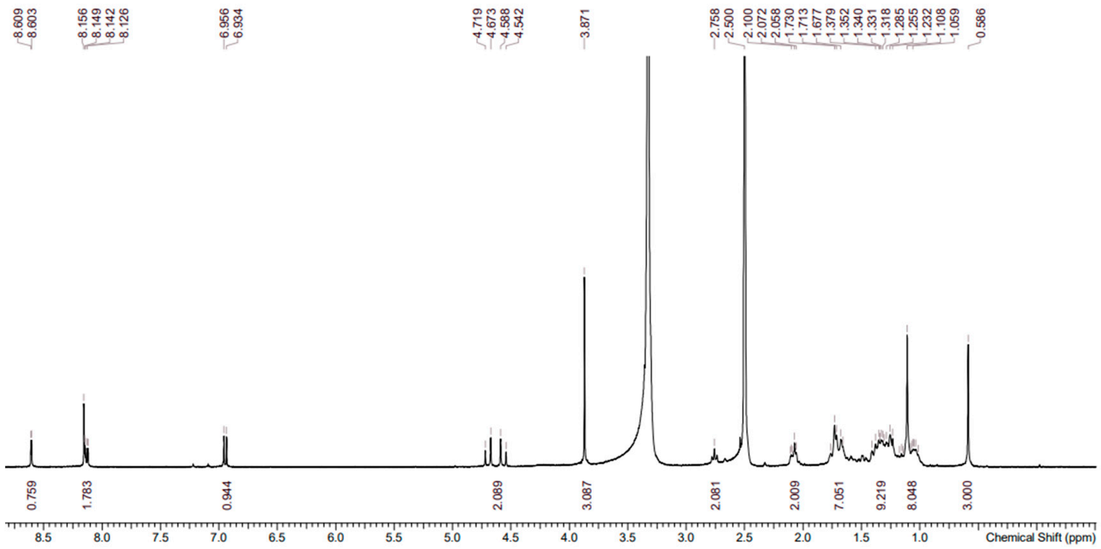

Figure S16. ^{1}H NMR (400 MHz, DMSO-d_6) spectrum of compound **S3**

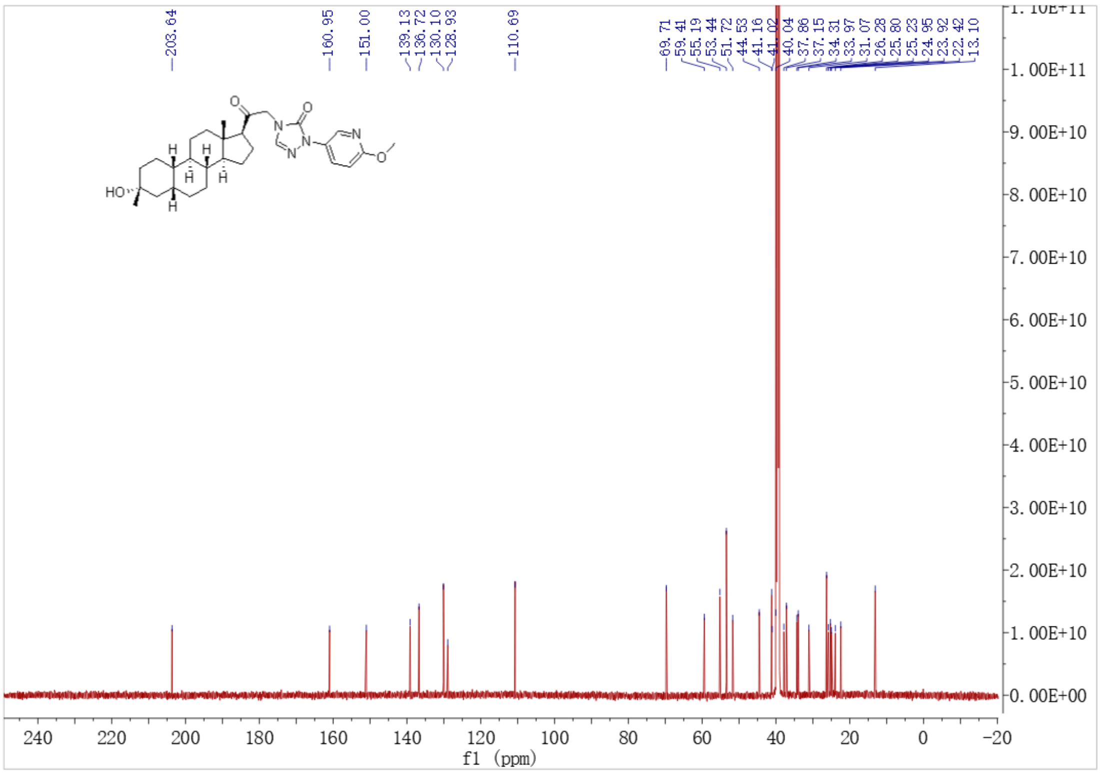

Figure S17. ^{13}C NMR (151 MHz, DMSO-d_6) spectrum of compound **S3**

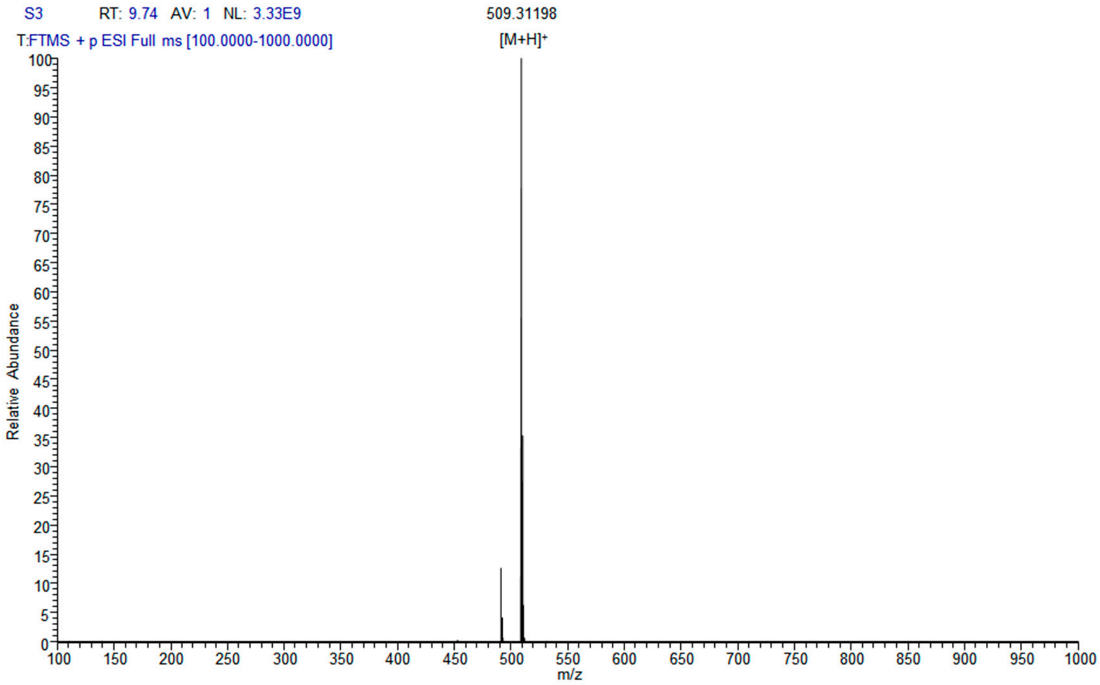

Figure S18. HRMS (ESI): m/z calcd for $C_{29}H_{41}N_4O_4$ $[M+H]^+$ 509.31223; found 509.31198.

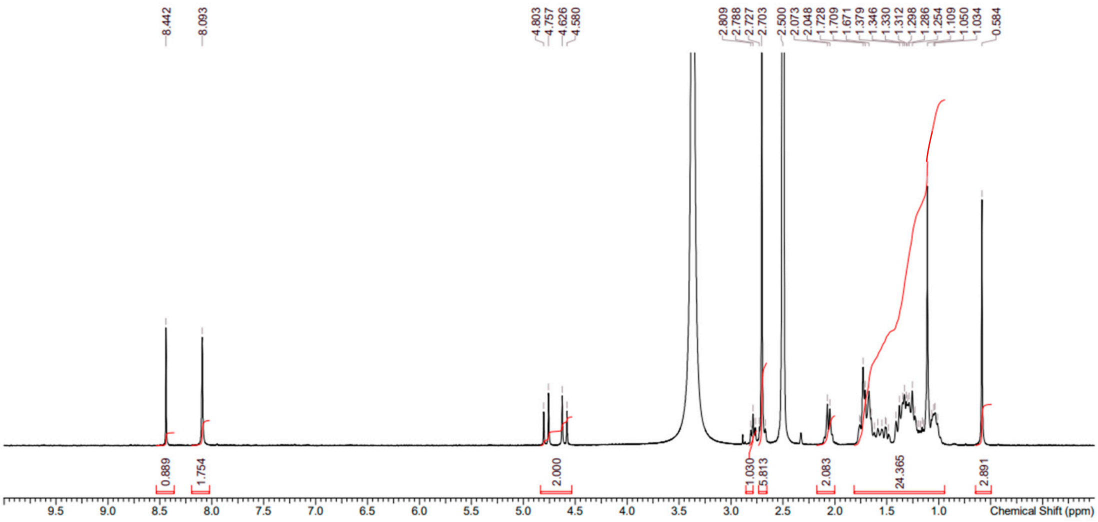

Figure S19. ^{1}H NMR (400 MHz, DMSO-d_6) spectrum of compound **S4**

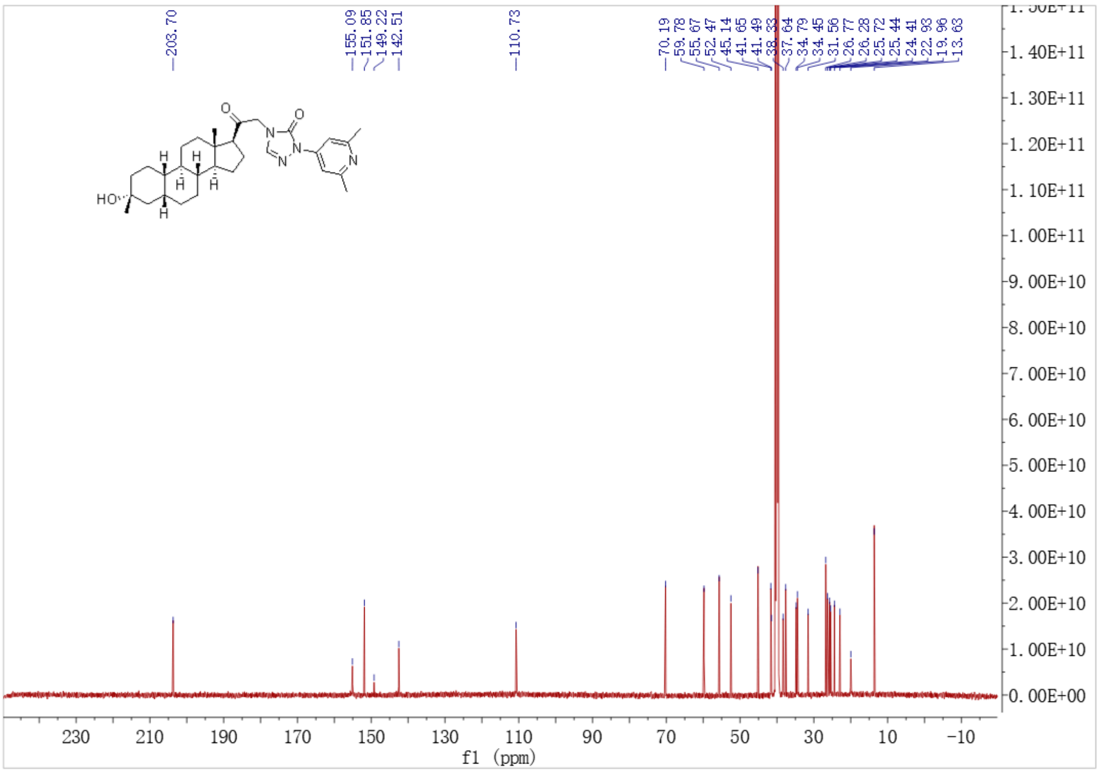

Figure S20. ^{13}C NMR (151 MHz, DMSO-d_6) spectrum of compound **S4**

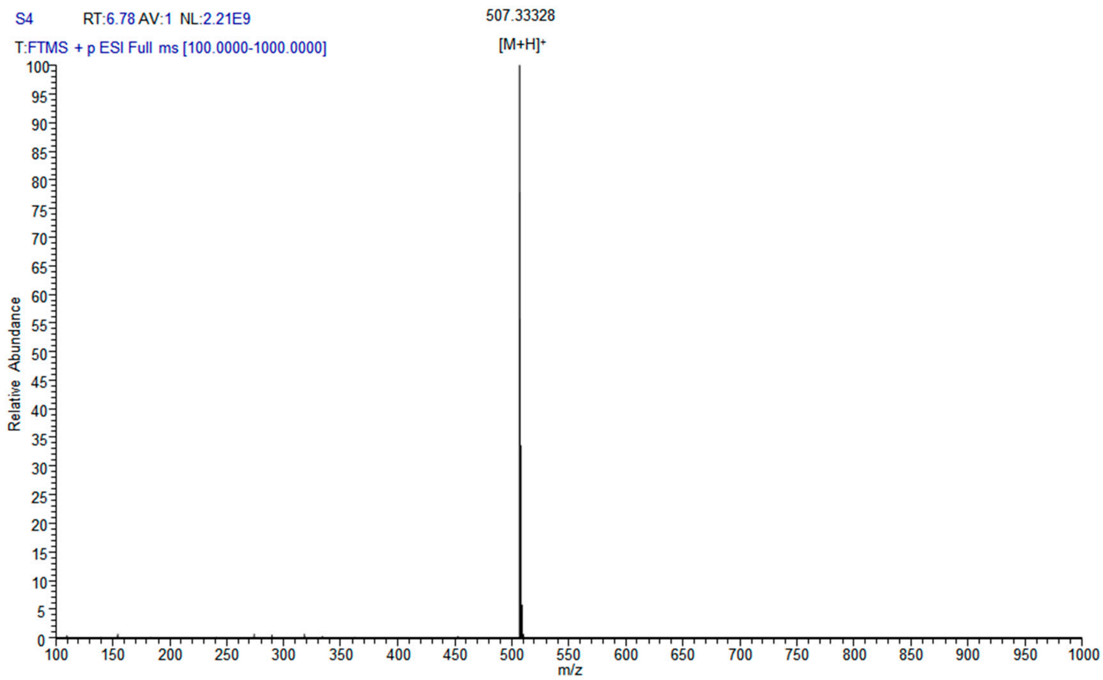

Figure S21. HRMS (ESI): m/z calcd for $C_{30}H_{43}N_4O_3$ $[M+H]^+$ 507.33297; found 507.33328.

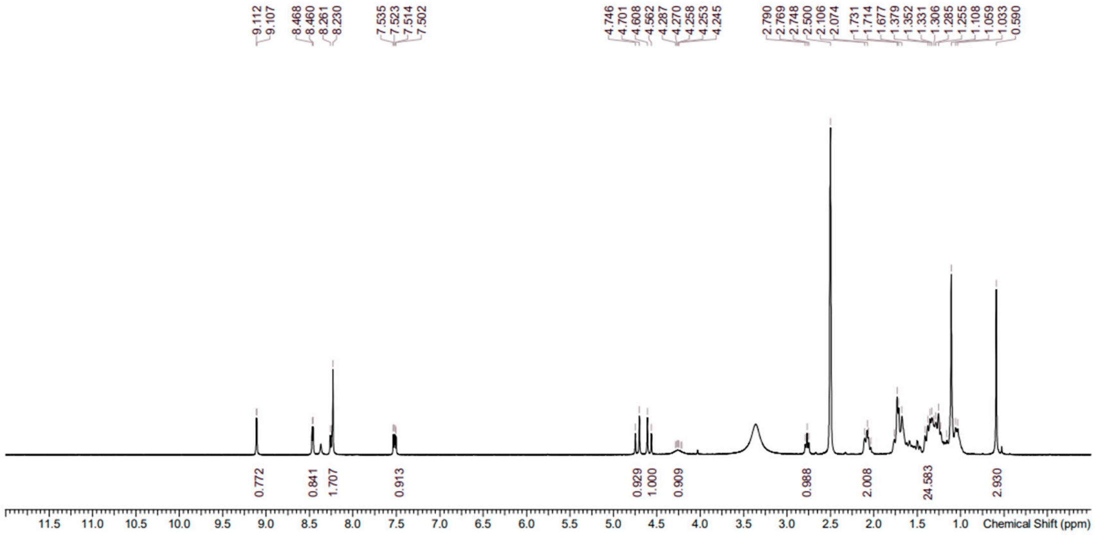

Figure S22. ^{1}H NMR (400 MHz, DMSO-d_6) spectrum of compound **S5**

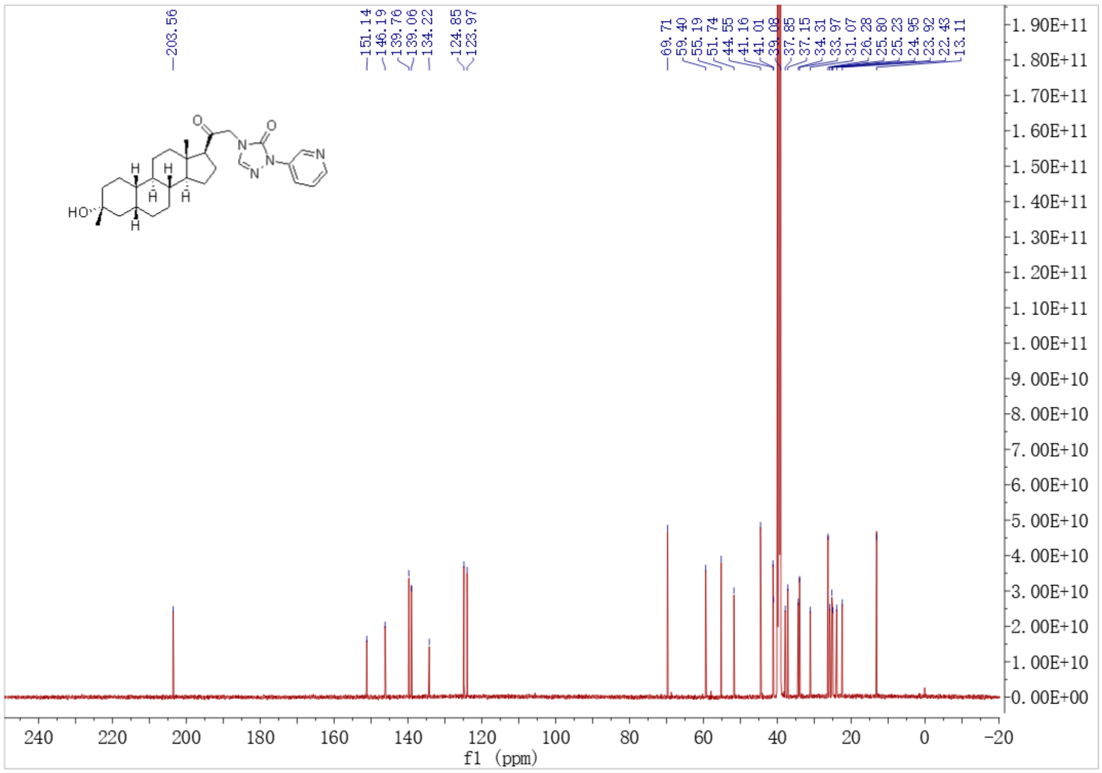

Figure S23. ^{13}C NMR (151 MHz, DMSO-d_6) spectrum of compound **S5**

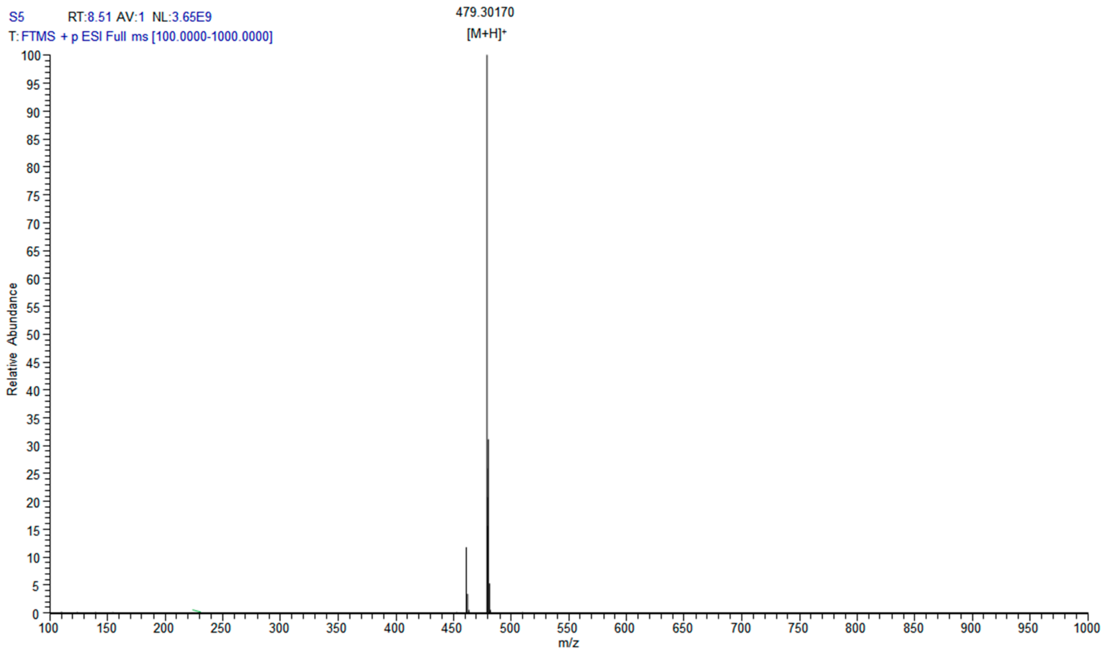

Figure S24. HRMS (ESI): m/z calcd for $C_{28}H_{39}N_4O_3$ $[M+H]^+$ 479.30167; found 479.30170.

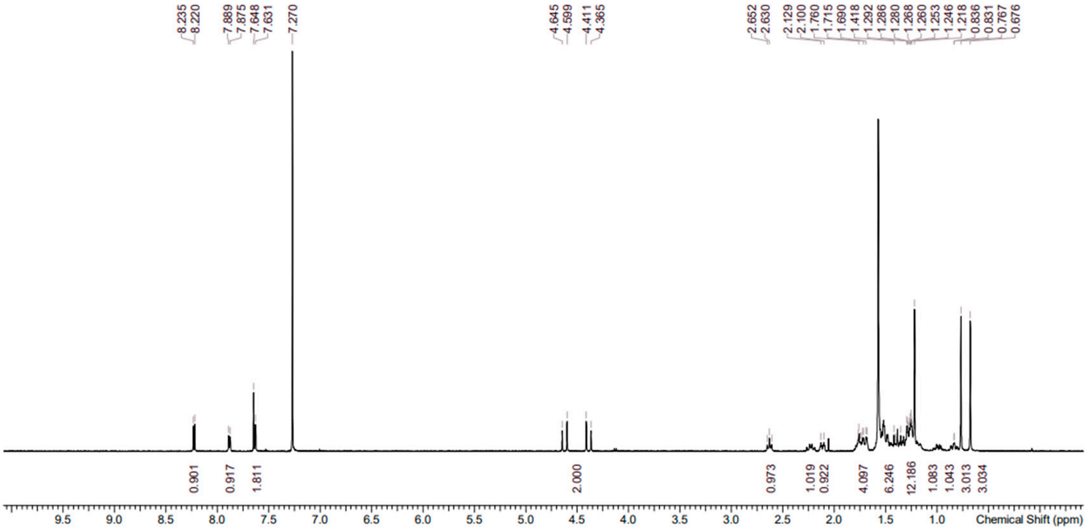

Figure S25. ^{1}H NMR (600 MHz, $CDCl_3$) spectrum of compound **S6**

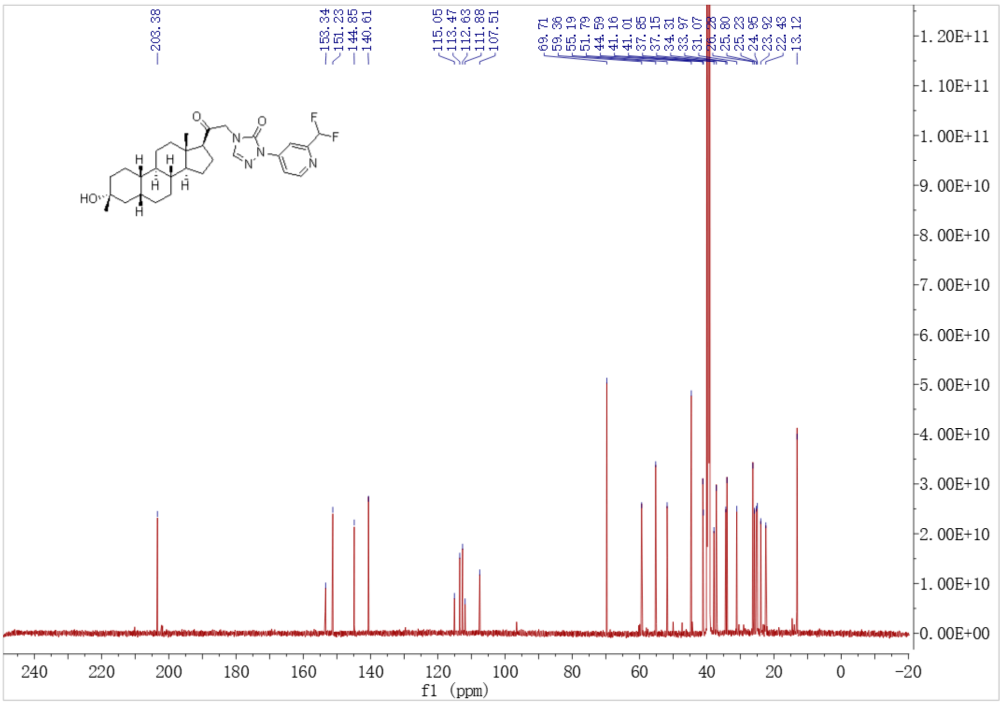

Figure S26. ^{13}C NMR (151 MHz, DMSO-d_6) spectrum of compound **S6**

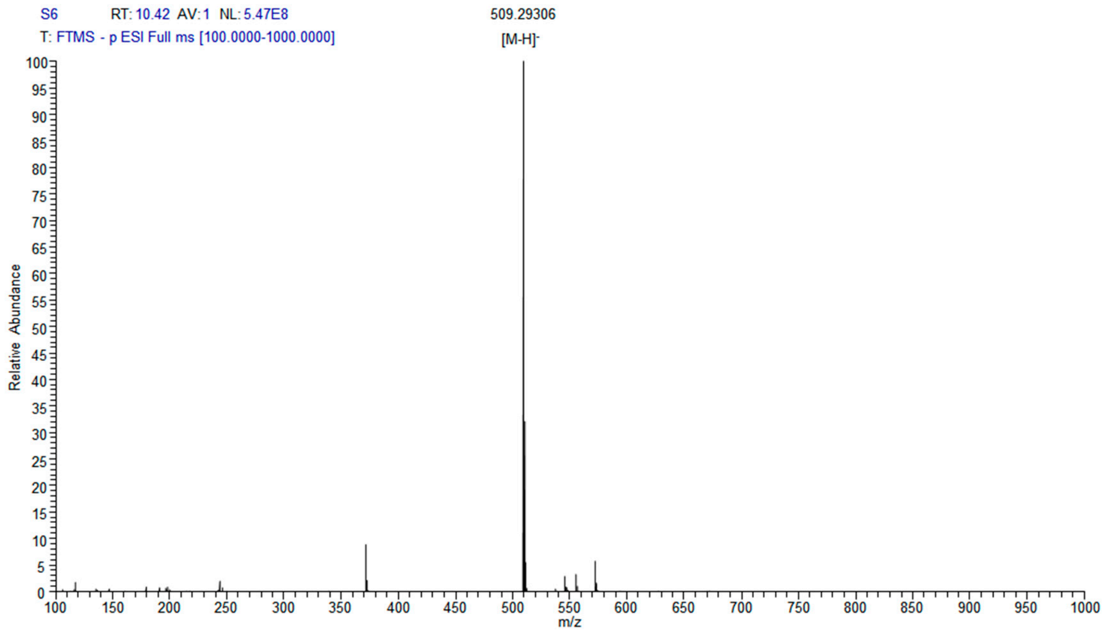

Figure S27. HRMS (ESI): m/z calcd for $C_{29}H_{38}FN_4O_3$ $[M-H]^-$ 509.29334; found 509.29306.

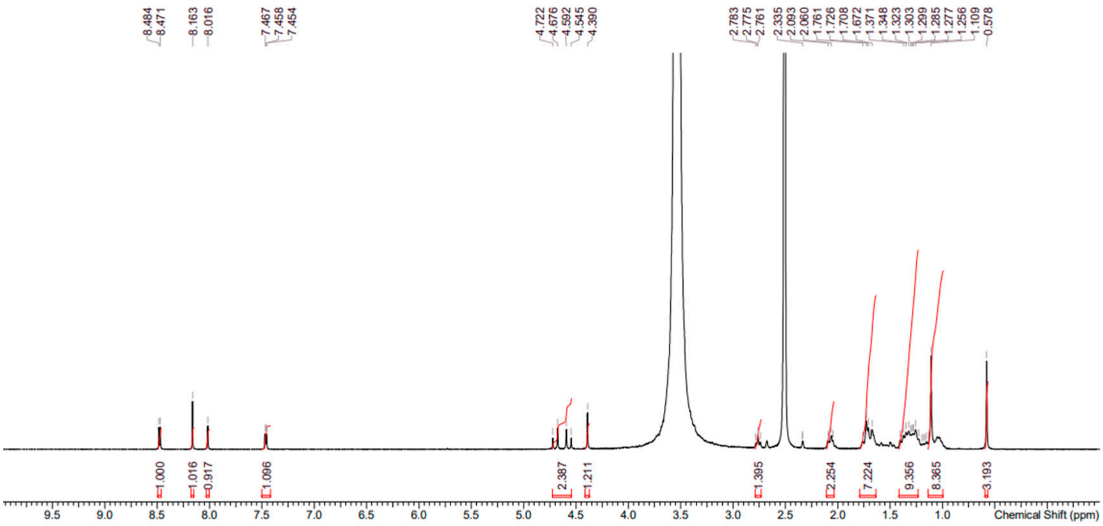

Figure S28. ^{1}H NMR (400 MHz, DMSO-d_6) spectrum of compound **S7**

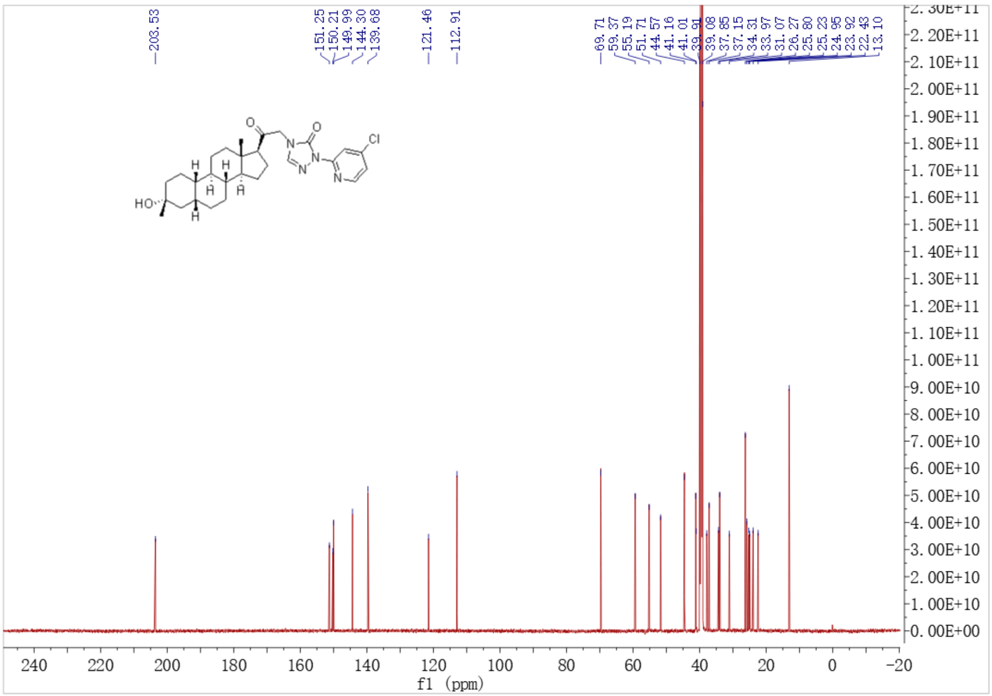

Figure S29. ^{13}C NMR (151 MHz, DMSO-d_6) spectrum of compound **S7**

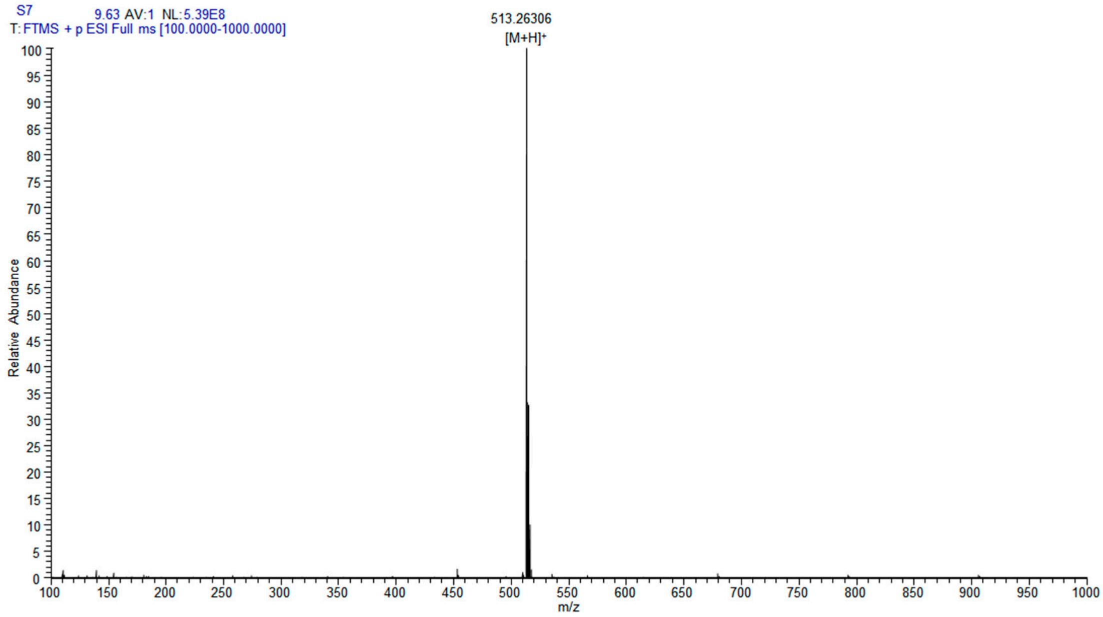

Figure S30. HRMS (ESI): m/z calcd for $C_{28}H_{38}ClN_4O_3$ $[M+H]^+$ 513.26270; found 513.26306.

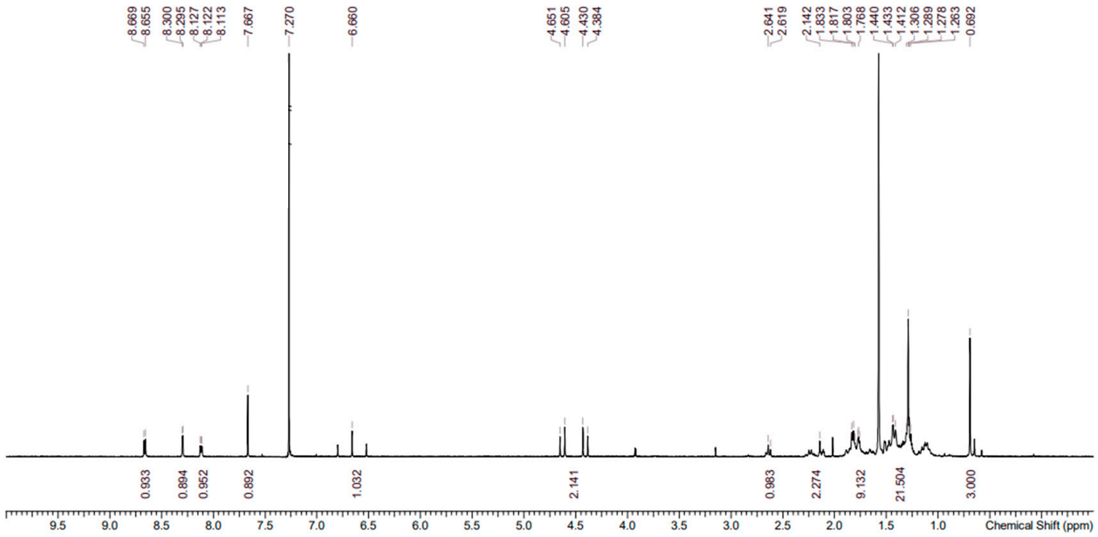

Figure S31. ^{1}H NMR (400 MHz, DMSO-d_6) spectrum of compound **S8**

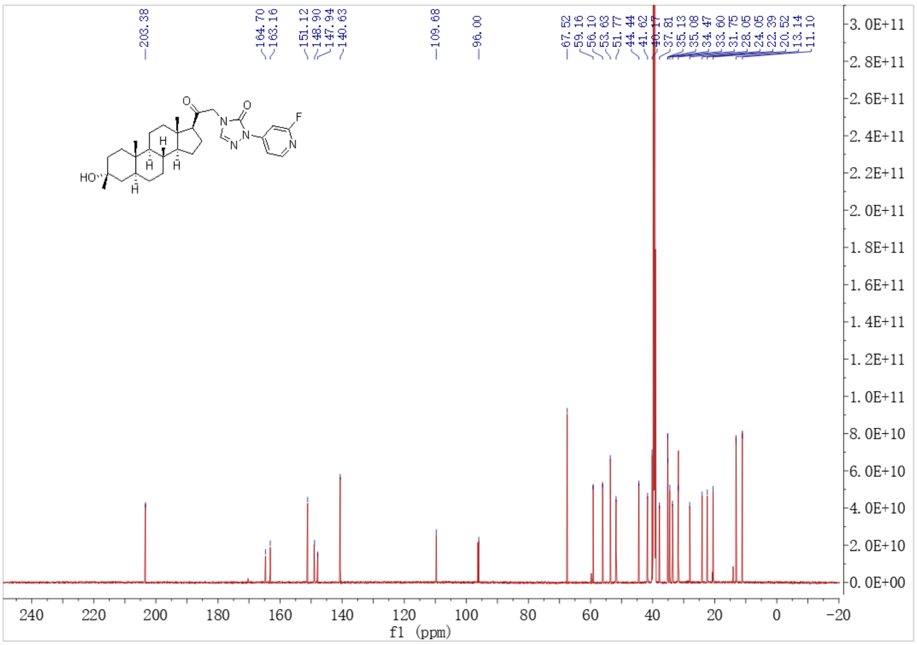

Figure S32. ^{13}C NMR (151 MHz, DMSO-d_6) spectrum of compound **S8**

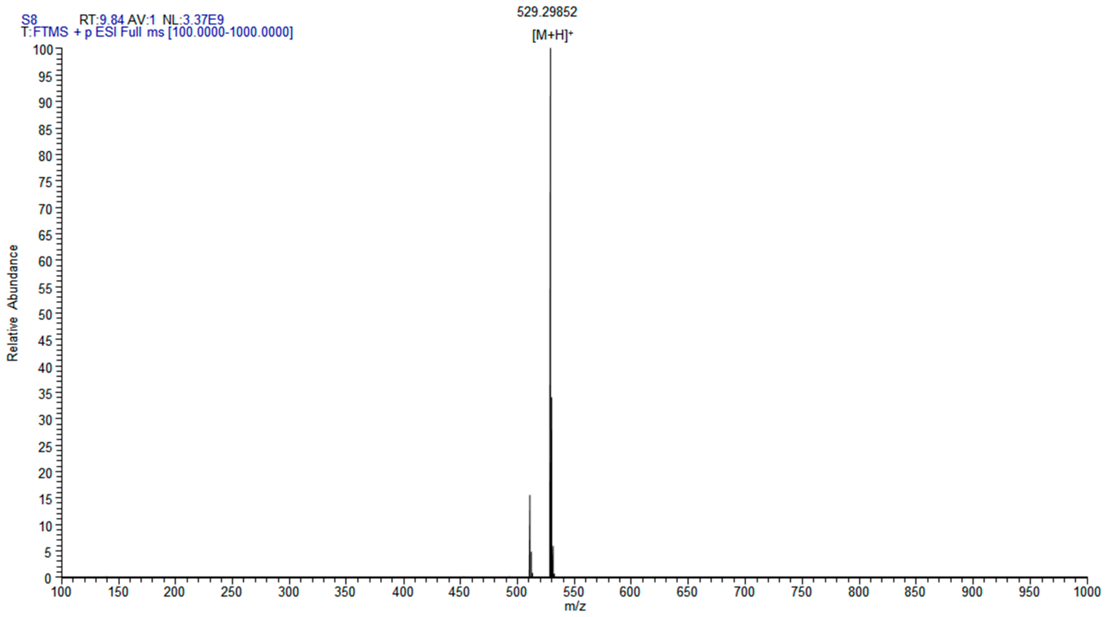

Figure S33. HRMS (ESI): m/z calcd for $C_{29}H_{39}F_2N_4O_3$ [M+H]$^+$ 529.29847; found 529.29852.

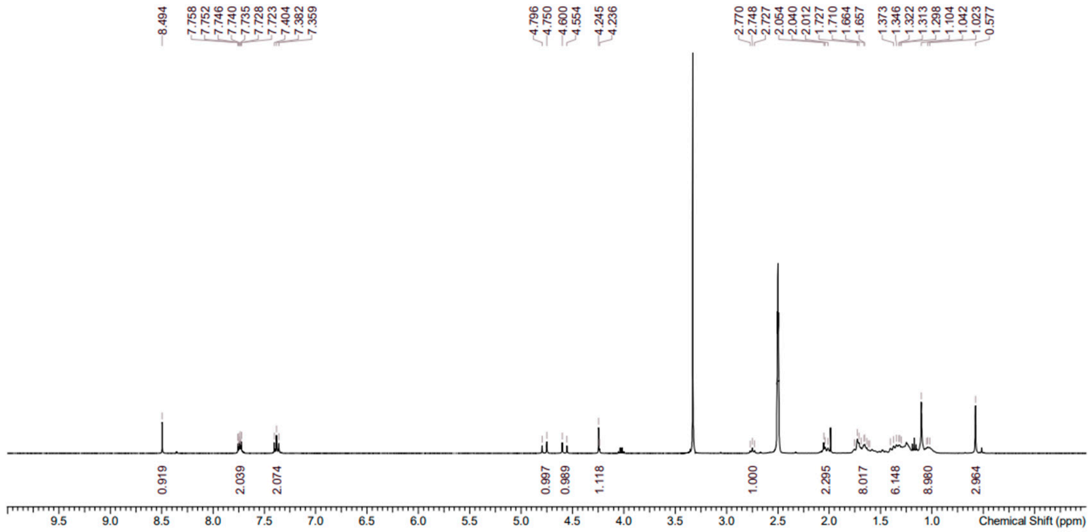

Figure S34. ^{1}H NMR (400 MHz, DMSO-d_6) spectrum of compound **S10**

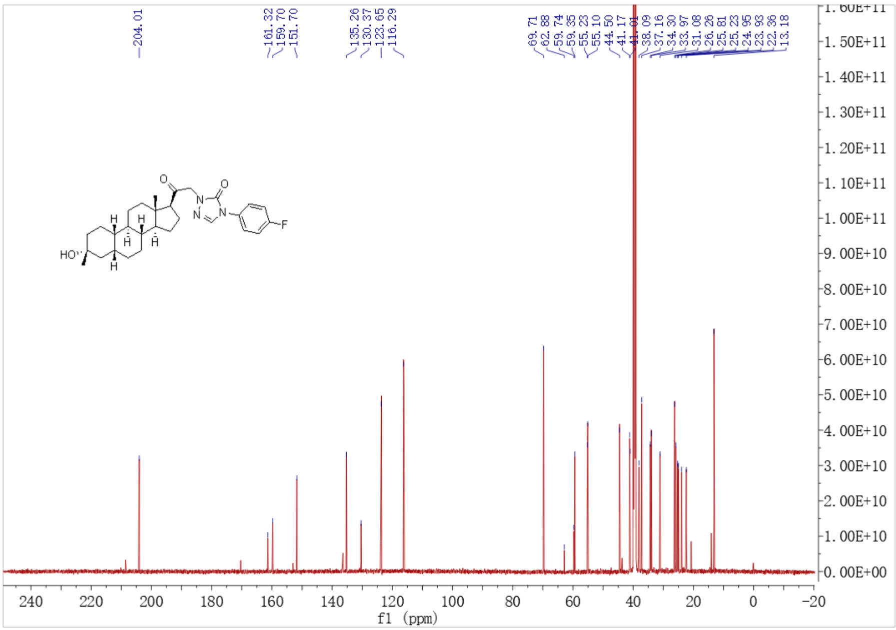

Figure S35. ^{13}C NMR (151 MHz, DMSO-d_6) spectrum of compound **S10** .

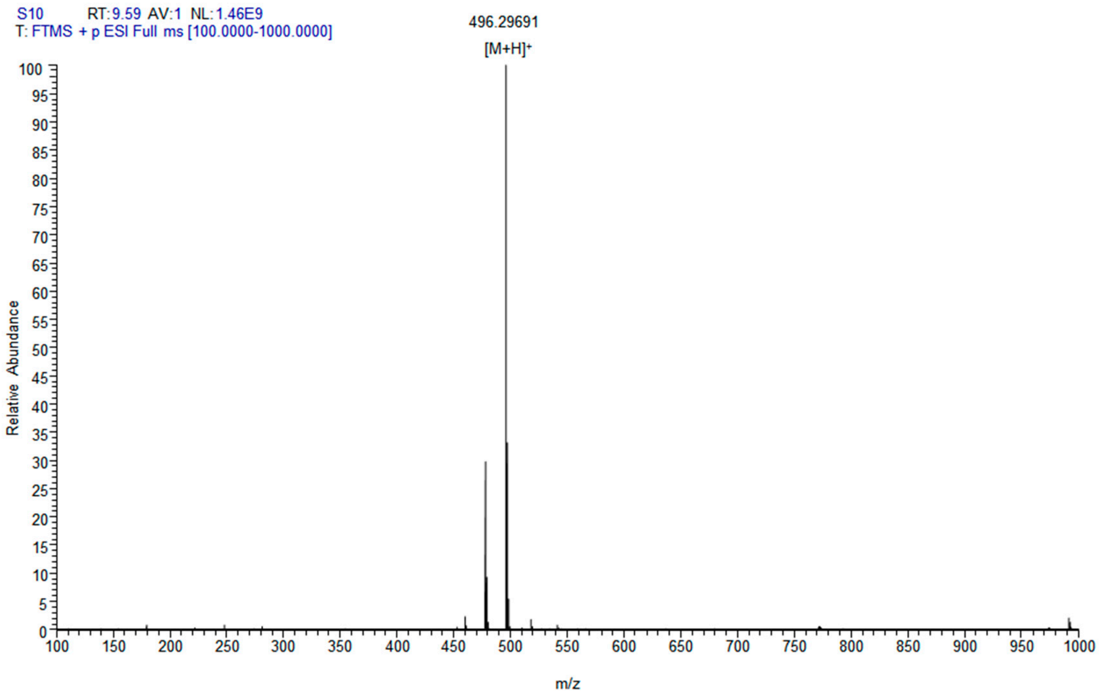

Figure S36. HRMS (ESI): m/z calcd for $C_{29}H_{39}FN_3O_3$ $[M+H]^+$ 496.29700; found 496.29691.

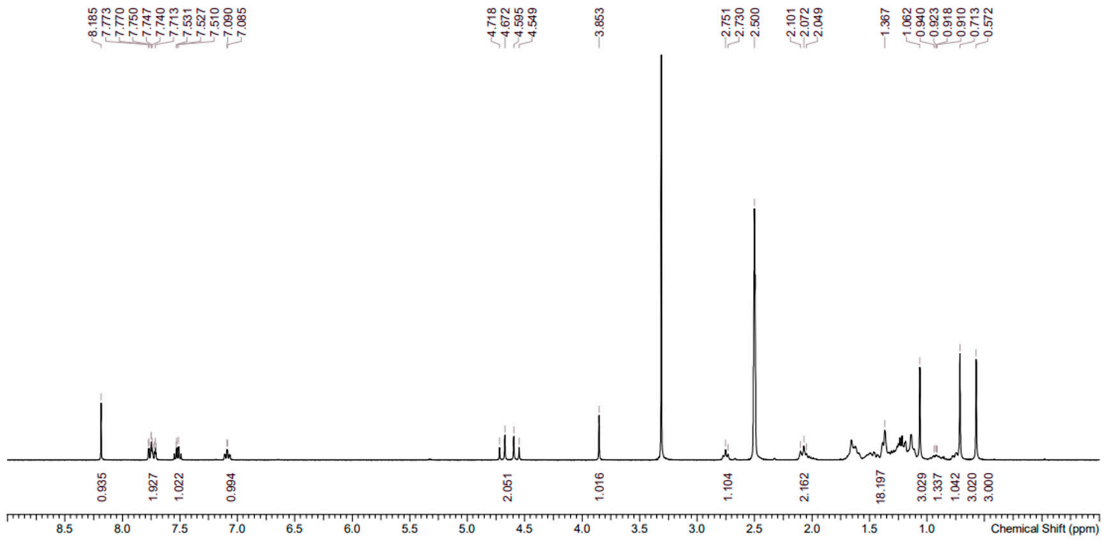

Figure S37. ^{1}H NMR (400 MHz, DMSO-d_6) spectrum of compound **S11**

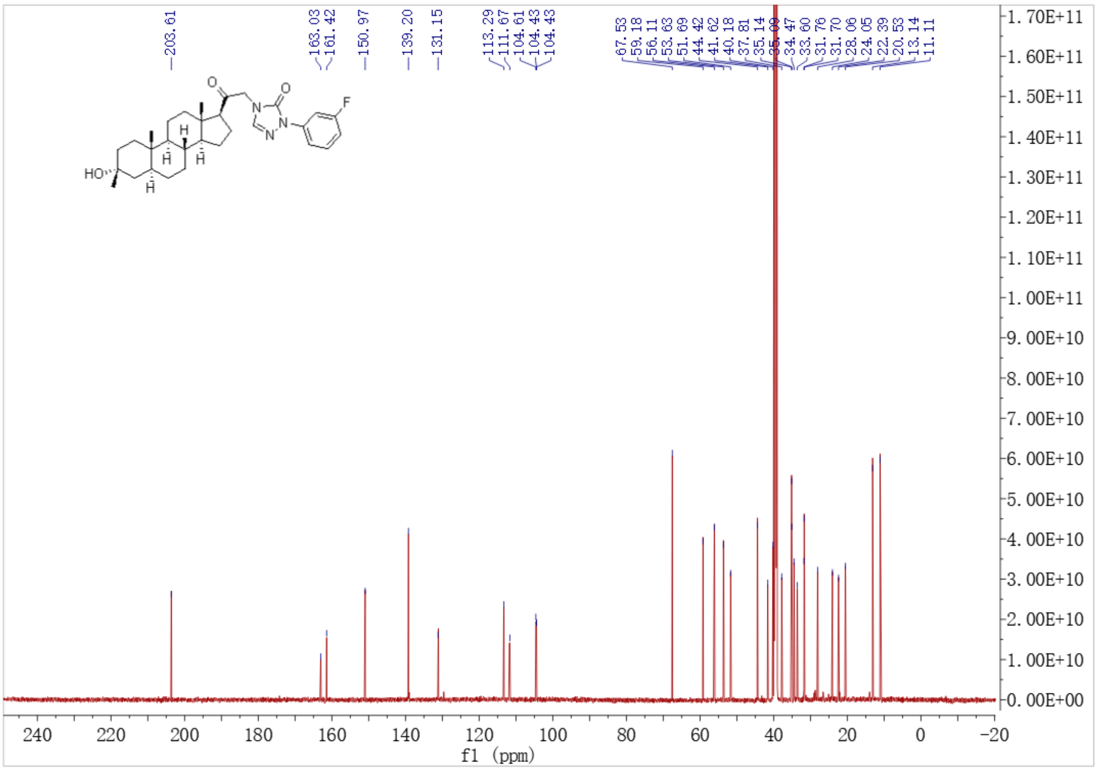

Figure S38. ^{13}C NMR (151 MHz, DMSO-d_6) spectrum of compound **S11**

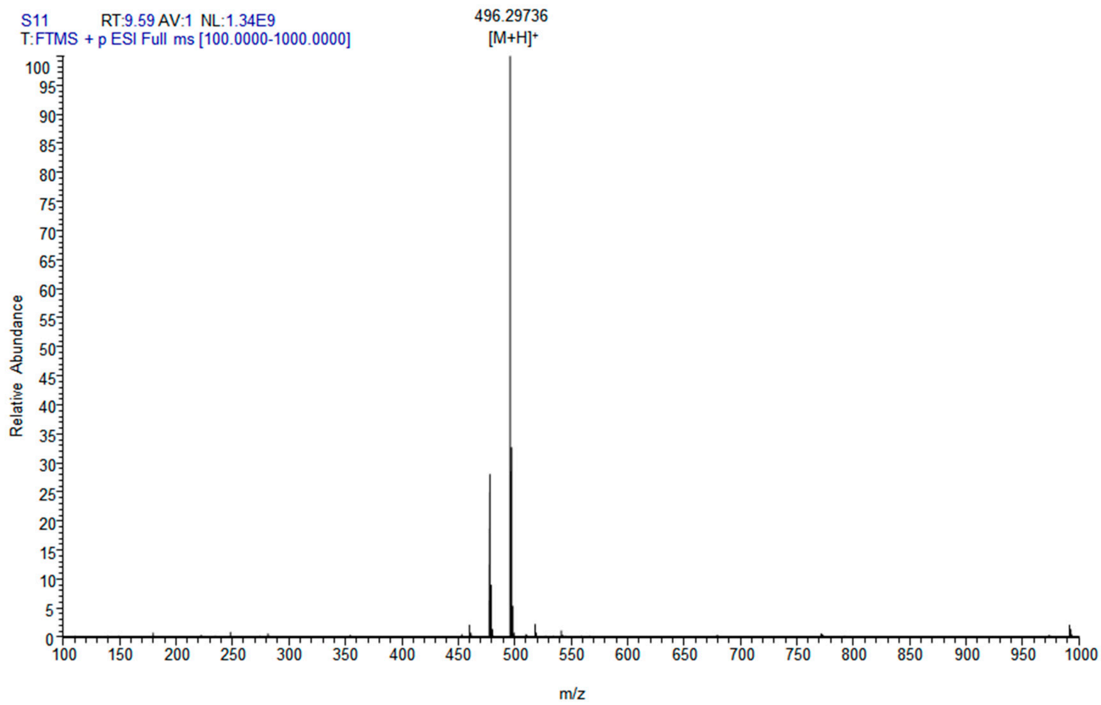

Figure S39. HRMS (ESI): m/z calcd for $C_{29}H_{39}FN_3O_3$ $[M+H]^+$ 496.29700; found 496.29736.

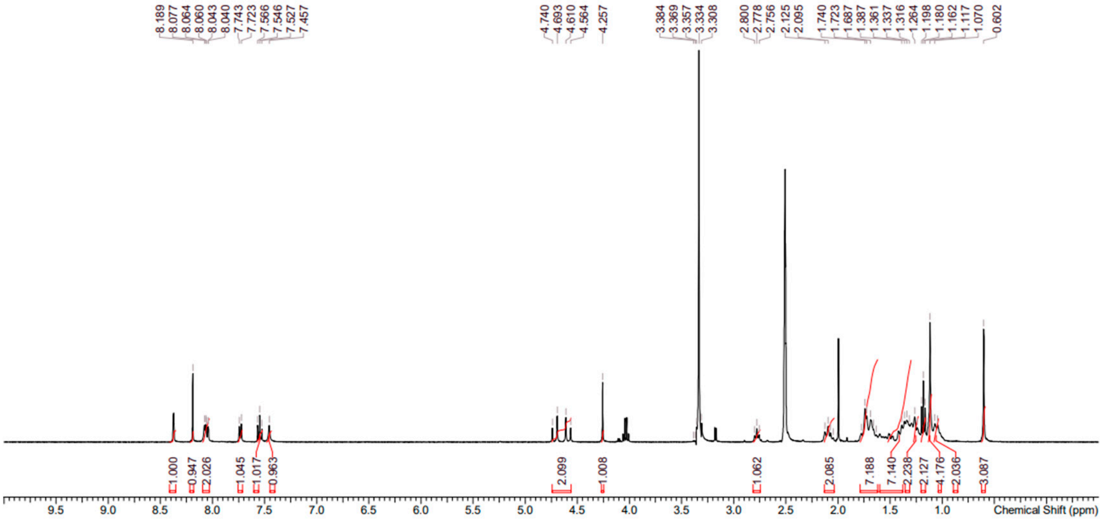

Figure S40. ^{1}H NMR (400 MHz, DMSO-d_6) spectrum of compound **S12**

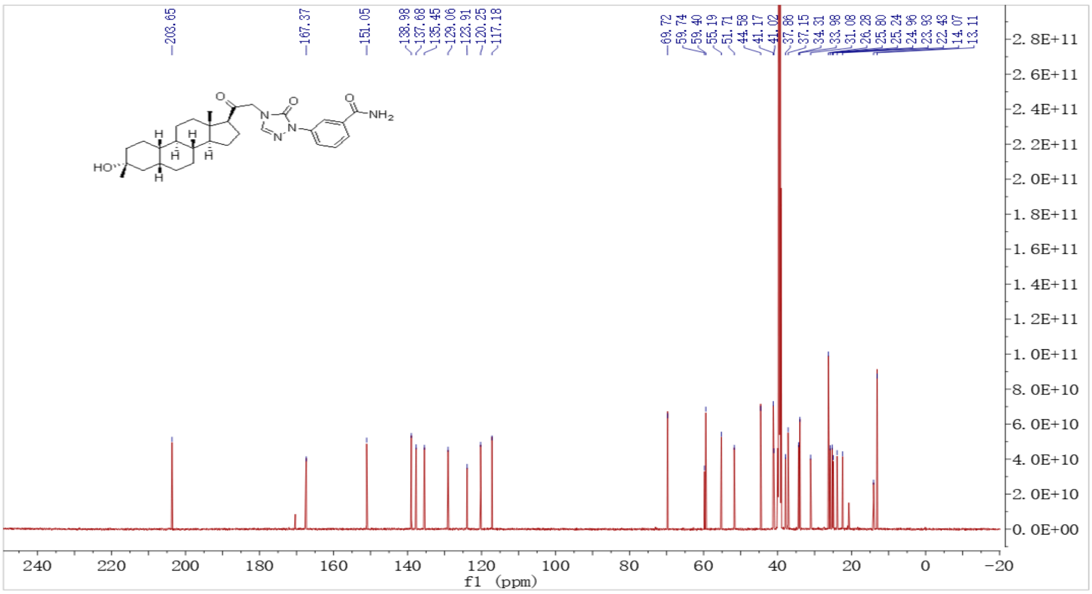

Figure S41. ^{13}C NMR (151 MHz, DMSO-d_6) spectrum of compound **S12**

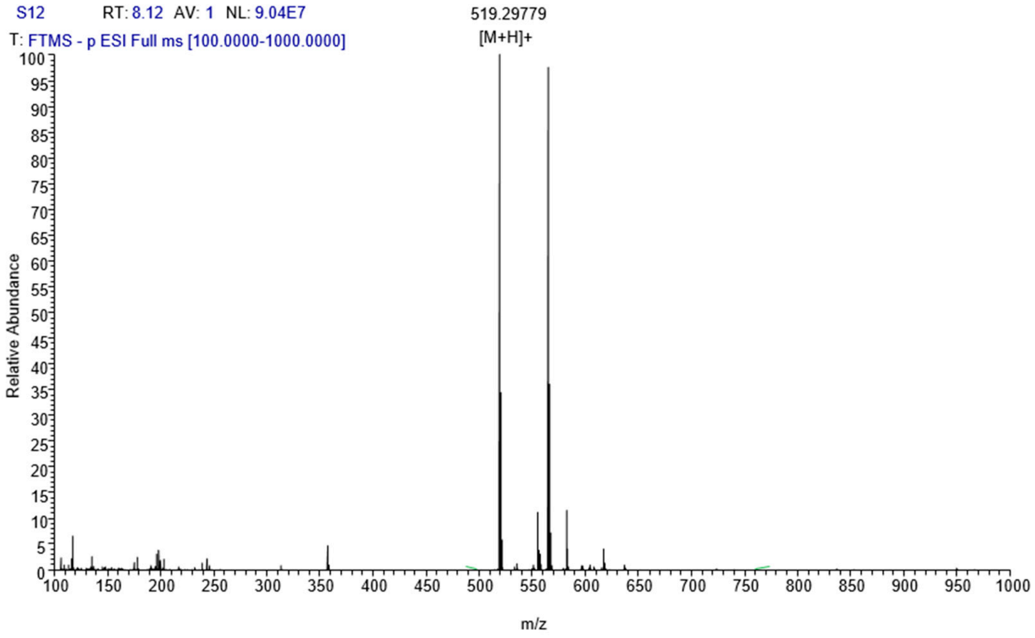

Figure S42. HRMS (ESI): m/z calcd for $C_{30}H_{39}N_4O_4$ $[M-H]^-$ 519.29768; found 519.29779.

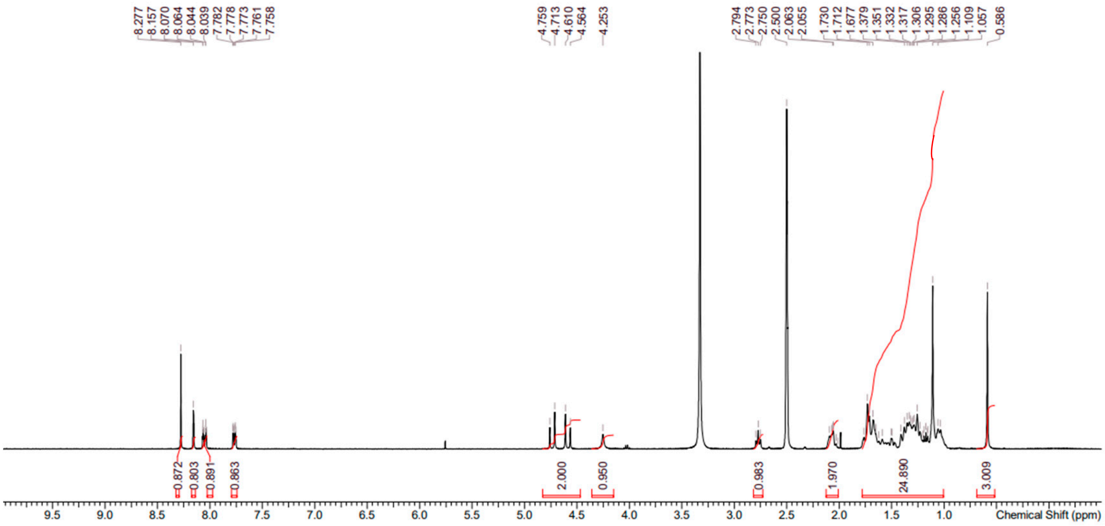

Figure S43. ^{1}H NMR (400 MHz, DMSO-d_6) spectrum of compound **S13**

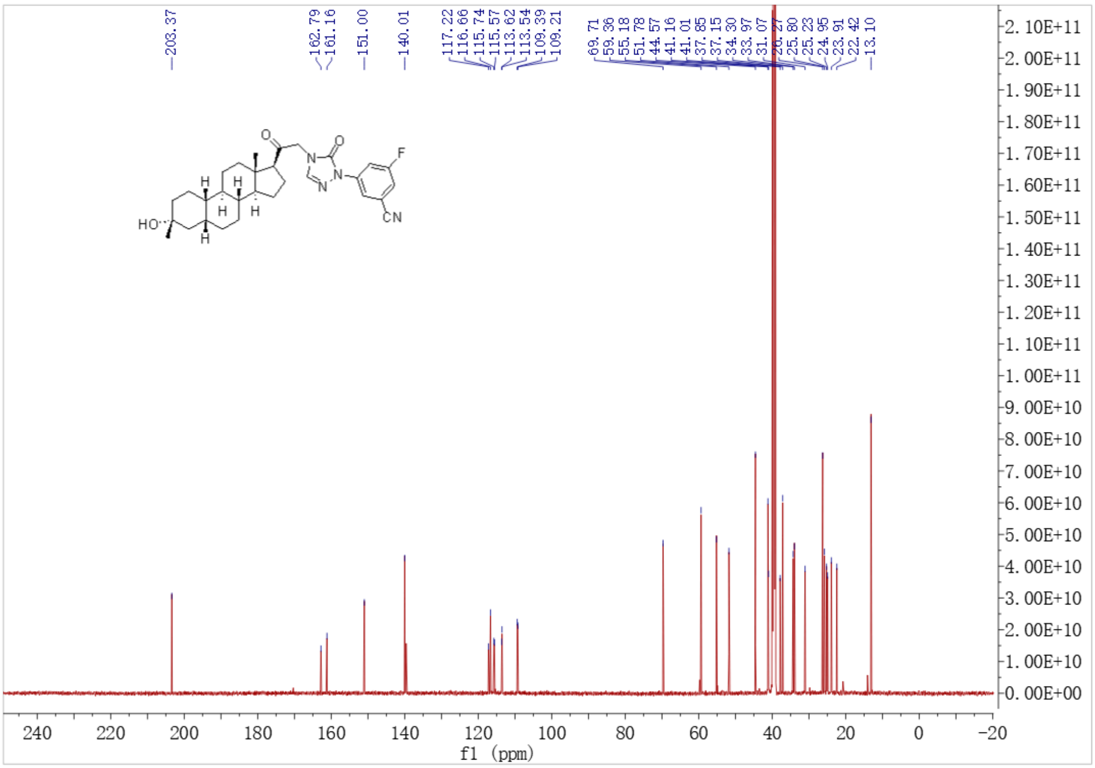

Figure S44. ^{13}C NMR (151 MHz, DMSO-d_6) spectrum of compound **S13**

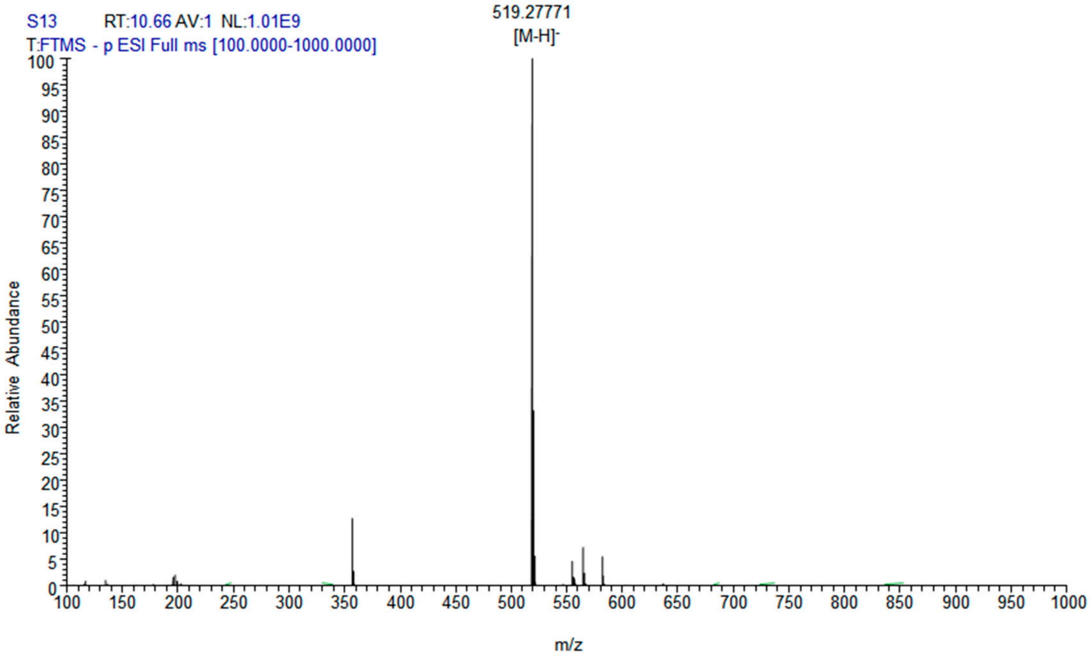

Figure S45. HRMS (ESI): m/z calcd for $C_{30}H_{36}FN_4O_3$ $[M-H]^-$ 519.27769; found 519.27771.

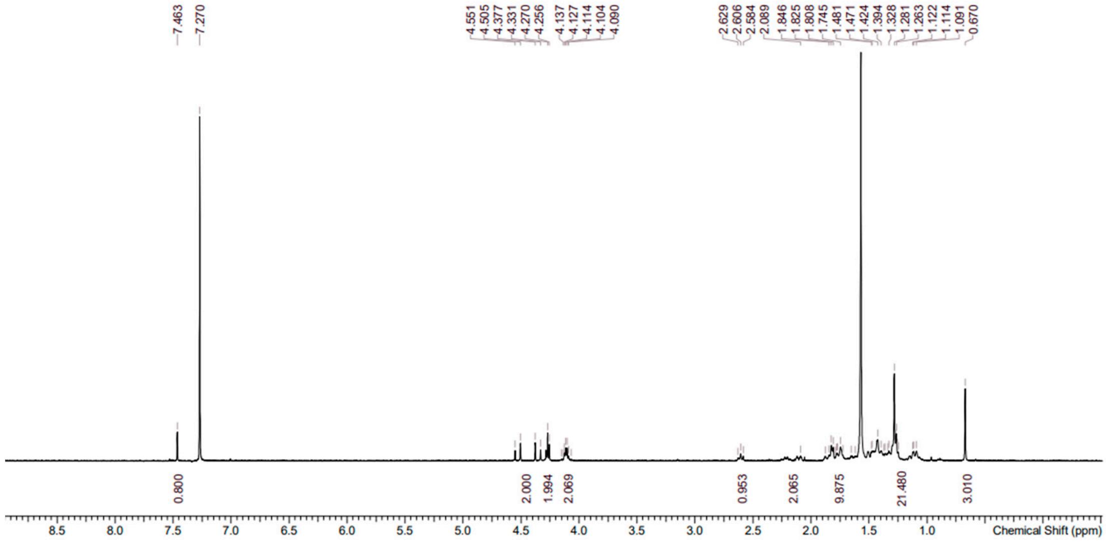

Figure S46. ^{1}H NMR (400 MHz, $CDCl_3$) spectrum of compound **S14**

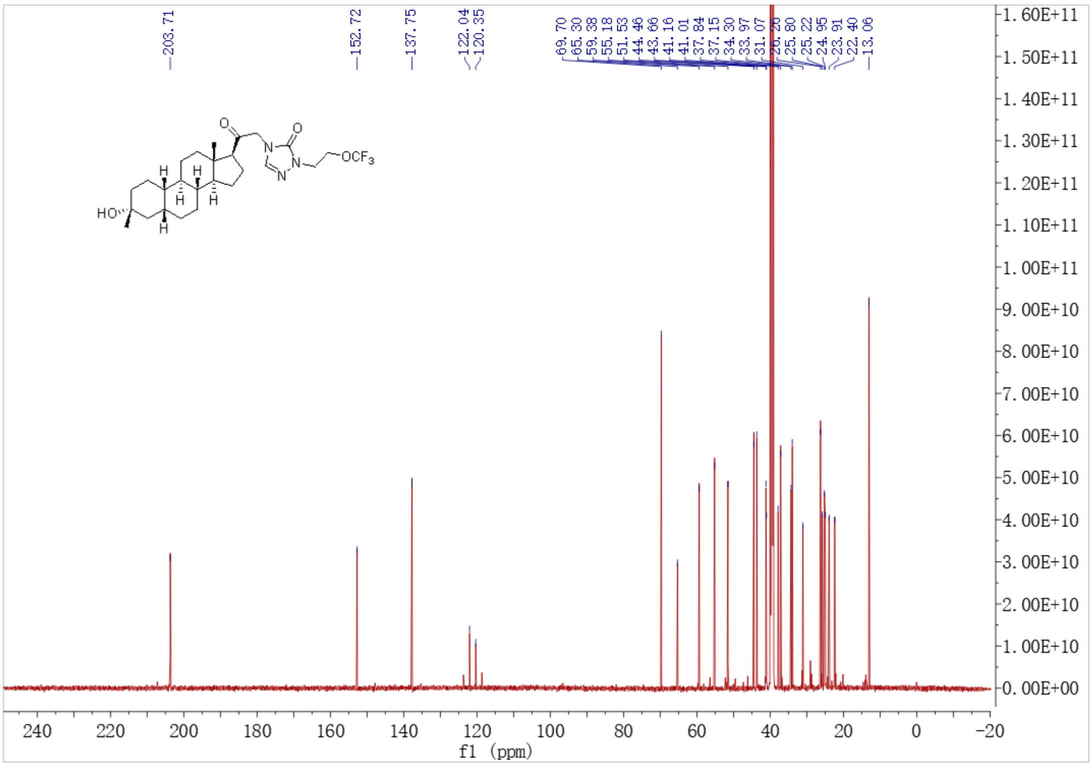

Figure S47. ^{13}C NMR (151 MHz, DMSO-d_6) spectrum of compound **S14**

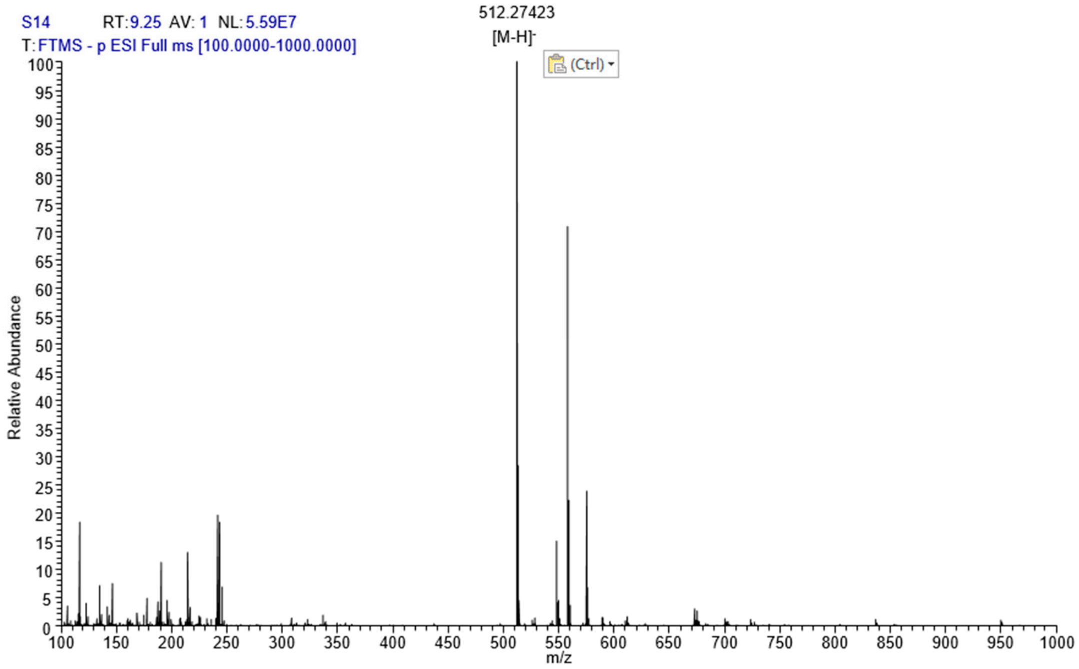

Figure S48. HRMS (ESI): m/z calcd for $C_{26}H_{37}F_3N_3O_4$ $[M-H]^-$ 512.27416; found 512.27423.

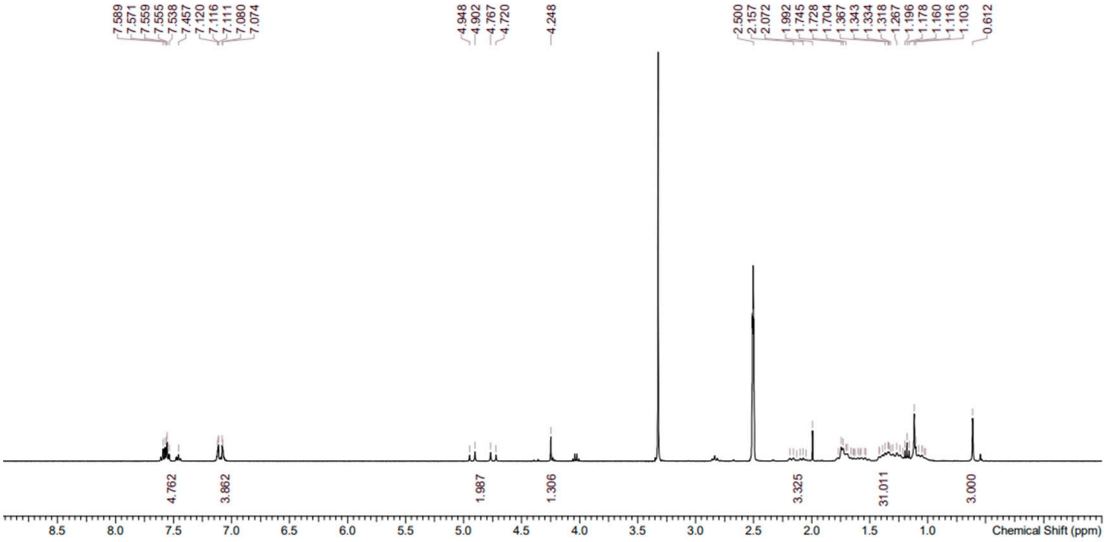

Figure S49. ^{1}H NMR (400 MHz, DMSO-d_6) spectrum of compound **S15**:

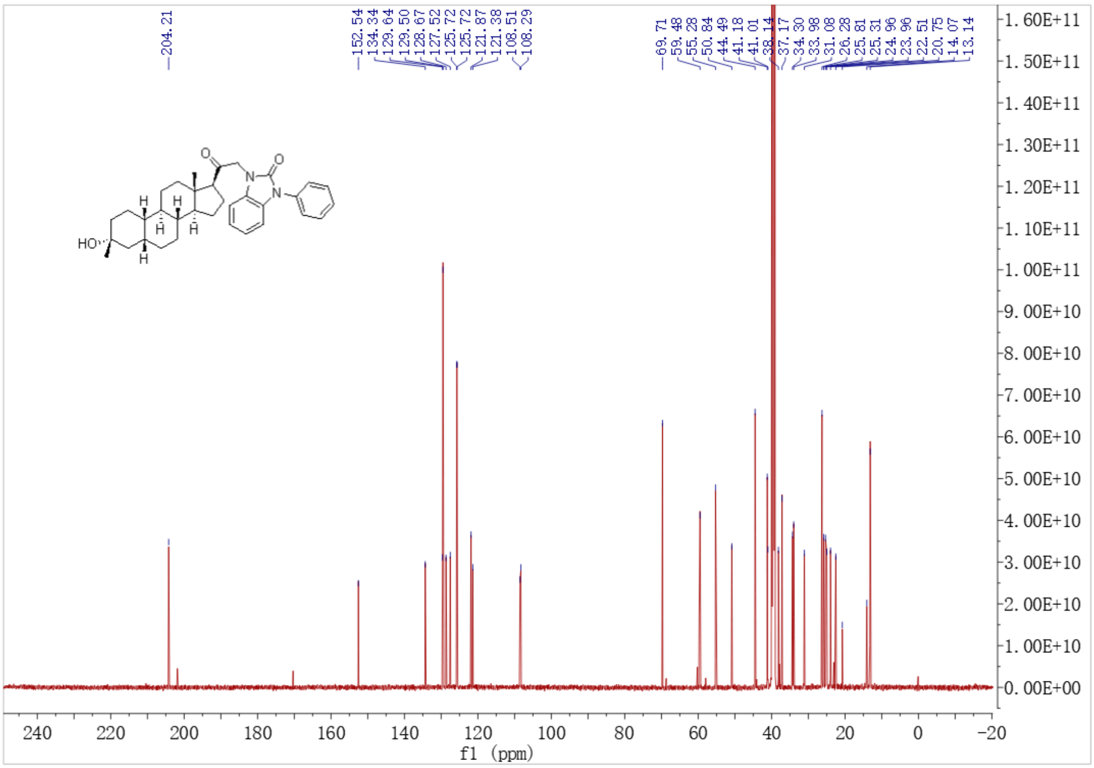

Figure S50. ^{13}C NMR (151 MHz, DMSO-d_6) spectrum of compound **S15**

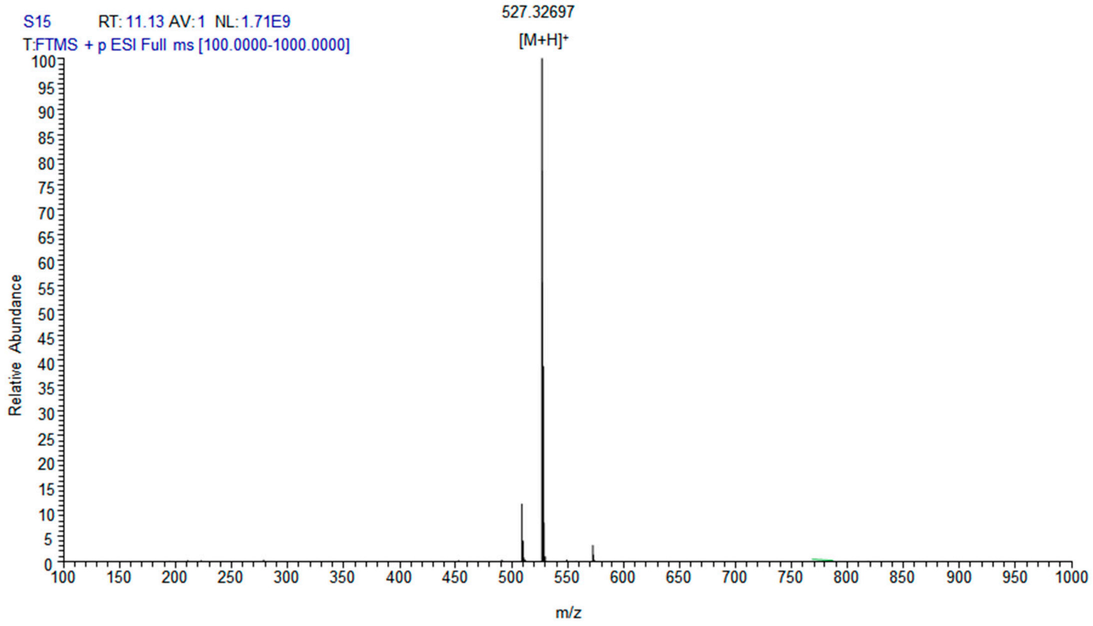

Figure S51. HRMS (ESI): m/z calcd for $C_{34}H_{43}N_2O_3$ $[M+H]^+$ 527.32682; found 527.32697.
